# K_2_CO_3_-Mediated Synthesis of Functionalised 4-Substituted-2-amino-3-cyano-4*H*-chromenes via Michael-Cyclization Reactions 

**DOI:** 10.3390/molecules191219253

**Published:** 2014-11-25

**Authors:** Yanyang He, Rong Hu, Rongsheng Tong, Fengqiong Li, Jianyou Shi, Mei Zhang

**Affiliations:** 1 Key Laboratory of Standardization of Chinese Herbal Medicines of Ministry of Education, State Key Laboratory Breeding Base of Systematic Research, Development and Utilization of Chinese Medicine Resources, School of Pharmacy, Chengdu University of Traditional Chinese Medicine, Chengdu 611137, China; E-Mails: heyanyang89@126.com (Y.H.); airinwalk@sohu.com (R.H.); l@lifq.cn (F.L.); 2 Pharmaceutical Department of Sichuan Academy of Medical Sciences, Sichuan Provincial People’s Hospital, Chengdu 610072, China; E-Mail: tongrs@126.com

**Keywords:** 2-amino-4*H*-chromenes, Michael-cyclization, malononitrile, K_2_CO_3_, Knoevenagel adducts, cascade reaction

## Abstract

An efficient approach for the synthesis of functionalized 4-substituted-2-amino-3-cyano-4*H*-chromenes moderate to high yields (up to 98%) has been achieved via a tandem K_2_CO_3 _catalyzed conjugate addition-cyclization reaction of malononitrile and a range of Knoevenagel adducts previously formed from oxindole, pyrazolone, nitromethane, *N,N*-dimethylbarbituric acid or indanedione. This methodology differs from the previous classical methods in its simplicity and ready availability of the catalyst.

## 1. Introduction

**Figure 1:**
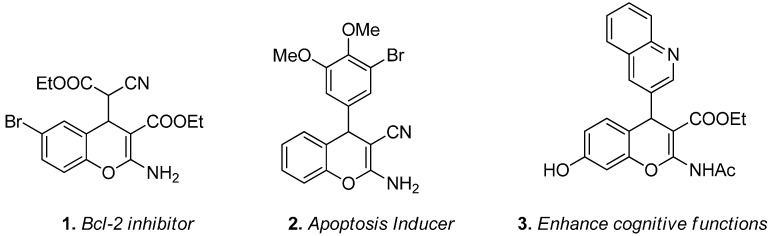
Selected examples of pharmacologically active compounds based on 4*H*-chromene scaffolds.

In recent years, the chromene ring moiety has emerged as a privileged scaffold for drug design and discovery because it exists in a myriad of biological natural products [1], pharmaceutical agents and drug candidates [2]. Chromene derivatives have attracted increasing attention from synthetic chemists due to their diverse biological activities, including antitumor [3], antibacterial [4], antiviral [5], antioxidative [6], antidepressant [7], antihypertensive [8], antidiabetic [9], fungicidal [10], and insecticidal properties [11]. In particular, among the various chromene derivatives, 2-amino-4*H*-chroemnes have been reported to exhibit highly useful proapoptotic properties for the treatment of a wide range of cancer ailments [12,13]. In cancer chemotherapy, 2-amino-4*H*-chromene **1** (Figure 1) was marked for drug development due to its high inhibition of tumor-associated Bcl-2 proteins [14]. The further modified 4*H*-chromene structure **2** (Figure 1) was able to induce apoptosis (programmed cell death) in several cancer cell lines [15]. 4-Aryl-4*H*-chromene **3** was found to have potential ability in the enhancement of cognitive functions, thus it is used in the treatment of neurodegenerative diseases [16]. For diversity oriented synthesis, the structure of these bioactive molecules could provide opportunities for drug design in three important regions (the aromatic ring of the benzopyran, substitution at the C2-amine, and the substituted group at the C4 position).

**Scheme 1:**
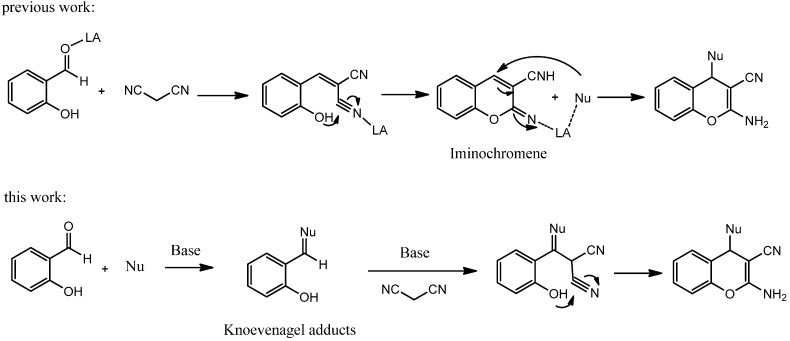
Strategies for the synthesis of substituted 2-amino-3-nitrile-4*H*-chromenes.

As a result, considerable efforts have been made over the past decades for the synthesis of 2-amino-4*H*-chromenes [17,18,19,20,21,22,23,24,25,26,27,28,29,30,31,32,33,34,35,36,37,38,39,40,41,42,43,44,45,46,47], which is accomplished using various catalysts including diethylamine [26], ethylenediamine diacetate [27], I_2_ [28], PEG [29], β-cyclodextrin [30], InCl_3_ [31,42,43], guanidine [32], ammonium acetate [46], Al_2_O_3_ [47], Zr(KPO_4_)_2_ [44], molecular sieves [45], aminosilane- modified Fe_3_O_4_ nanoparticles [33] and silica-bonded 2-hydroxyethylammonium acetate (HEAA) [34]. In addition, some enantioselective synthesis methodologies for 2-amino-4*H*-chromenes were also documented in the literature [22,23,35,36,37,38,39]. However, these methods show varying degrees of success as well as limitations, such as requiring complex and expensive catalytic systems, prolonged reaction times and complicated operations. Therefore, it is still deemed worthwhile and important to explore the direct use of an inexpensive and readily available organic species as catalyst for the above synthesis. The most straightforward synthesis of this heterocyclic nucleus involves the MCR of salicylaldehyde, malononitrile and nucleophiles, which can be catalyzed by various Lewis acids [22,23,31,42,43,44,47] (Scheme 1, previous work). It is worth mentioning that iminochromene was first formed, and then reacted with diverse C-, N-, S-, P-nucleophiles to give a wide range of substituted and fused chromenes. As part of our efforts toward the efficient synthesis of 2-amino-3-nitrile-4*H*-chromenes, we decided to seek a new class of readily available starting materials. A designed Michael addition triggered cascade reaction of malononitrile with Knoevenagel adducts generated from salicylaldehyde and nucleophiles was tested (Scheme 1, this work).

Therefore, in continuation of our interest in synthetic strategies for the preparation of heterocyclic compounds, a new K_2_CO_3_-catalyzed methodology for the synthesis of diverse 4-substituted-2-amino-3-cyano-4*H*-chromenes bearing various substituent groups at the C4 position was developed. These substituents are the most intensively studied structural motifs, and crucial building blocks for the synthesis of biologically active compounds and natural products [23] as key synthons in planning the synthesis of therapeutic agents and exhibiting diverse pharmaceutical activities.

## 2. Results and Discussion

Initially, the reaction of 3-(2-hydroxybenzylidene)-indolin-2-one (**4a**) and malononitrile (**5**) in THF was tested with different bases under mild conditions. Most organic bases such as DBU, piperidine, DIPEA and Et_3_N showed no activity within 2 h in this reaction (Scheme 1 and Table 1, entries 1–4). With the prolonged time, only a small amount of product was formed. Other organic bases such as DMAP and DABCO could promote the reaction and afforded the product **6a** in low yield (31% and 13%, respectively, entries 5, 6). However, when the catalyst was replaced with an inorganic base, the reaction proceeded efficiently and was complete in less than 2 h at ambient temperature (Table 1, entries 7,8). Encouraged by the above results, more efforts were made to optimize other reaction parameters including solvents and reaction temperatures. Thus, the reaction was studied in different solvents that included THF, CH_3_OH, C_2_H_5_OH, CHCl_3_, CH_2_Cl_2_, H_2_O, toluene, dioxane, CH_3_CN and DMF (Table 1, entries 8–16). It was found that THF gave comparable yields (Table 1, entry 8), but other polar solvents, such as DMF, DMSO and water could not promote the reaction (Table 1, entries 11,13,14). The temperature also influenced the rate of the reaction. Increasing the reaction temperature resulted in a high reactivity (Table 1, entries 18–21), and conducting the reaction at 60 °C provided the best results. Notably, the two diastereoisomers of **6a** could be easily isolated by silica gel chromatography and the diastereomer ratio was 1:1. Based on the comprehensive consideration of reaction temperature and yield, the optimal reaction conditions were established as shown in Table 1, entry 19. The ratio of two diastereomers remained the same in all the cases.

**Table 1:** Optimization for the synthesis of 2-amino-4-(2-oxoindolin-3-yl)-4*H*-chromene **6a** ^a^.

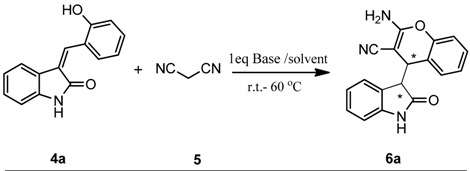

Entry	Solvent	Base	Temp.	Time	Yield of 6a ^b,c^
1	THF	DBU	r.t.	2 h	0
2	THF	Piperidine	r.t.	2 h	0
3	THF	DIPEA	r.t.	2 h	0
4	THF	Et_3_N	r.t.	2 h	0
5	THF	DMAP	r.t.	2 h	31%
6	THF	DABCO	r.t.	2 h	13%
7	THF	Na_2_CO_3_	r.t.	2 h	61%
8	THF	K_2_CO_3_	r.t.	2 h	95%
9	CH_3_OH	K_2_CO_3_	r.t.	2 h	45%
10	C_2_H_5_OH	K_2_CO_3_	r.t.	2 h	44%
11	CH_3_Cl	K_2_CO_3_	r.t.	2 h	34%
12	CH_2_Cl_2_	K_2_CO_3_	r.t.	2 h	35%
13	H_2_O	K_2_CO_3_	r.t.	2 h	0
14	Toluene	K_2_CO_3_	r.t.	2 h	40%
15	Dioxane	K_2_CO_3_	r.t.	2 h	0
16	DMF	K_2_CO_3_	r.t.	2 h	0
17	CH_3_CN	K_2_CO_3_	r.t.	2 h	66%
18	THF	K_2_CO_3_	40 °C	50 min	95%
19	THF	K_2_CO_3_	60 °C	10 min	98%
20	CH_3_CN	K_2_CO_3_	40 °C	35 min	91%
21	CH_3_CN	K_2_CO_3_	60 °C	7 min	98%

^a ^Reaction conditions: **4a** (0.1 mmol), **5** (0.1 mmol), Base (0.1 mmol) in solvent (0.5 mL). ^b^ Isolated yield after silica gel chromatography. ^c^ dr = 1:1 calculated from the isolated isomers.

After having established the optimal conditions for the synthesis of 2-amino-4-(2-oxoindolin-3-yl)-4*H*-chromene, the scope of reaction were explored with various Knoevenagel adducts derived from oxindole (Table 2). For the substrates bearing electron-donating (-Me) and electron-withdrawing groups (-Cl, -F) on the indole ring, the reactions proceeded smoothly to give the corresponding substituted 4*H*-chromenes **6** in 83%–98% yields. However, the Knoevenagel adducts with *o*-, *m*-substituents gave lower activity than those with *p*-substituents. In addition, the 3-F group (Table 2, entry 6) substrate gave a complex reaction mixture at 60 °C. Lowering the reaction temperature to room temperature, **6f** was obtained in 85% yield by prolonging the reaction time. The diastereoselectivity of almost all of these reactions (except entry 13) was 1:1 and the two diastereoisomers could be isolated by silica gel chromatography. Thus, equivalent diastereoisomers were easily obtained in one step. This transformation was also suitable for ethyl cyanoacetate, furnishing compound **6l** in 67% yields with higher diastereoselectivity (10:1). 

**Table 2:** Synthesis of new 2-amino-4-(2-oxoindolin-3-yl)-4*H*-chromene **6a**–**m** ^a^.

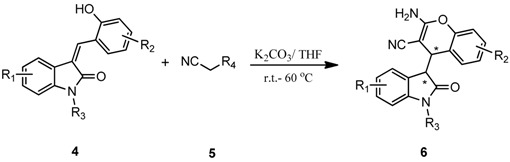

Entry	R_1_	R_2_	R_3_	R_4_	Product 6	Time	Yield ^b,c^
1	H	H	H	CN	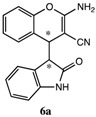	10 min	98%
2	H	5-CH_3_	H	CN	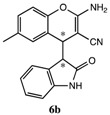	8 min	90%
3	H	5-Cl	H	CN	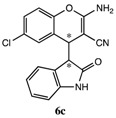	8 min	95%
4	6-Cl	H	H	CN	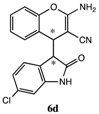	6 min	93%
5	H	5-Br	H	CN	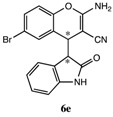	6 min	91%
6 ^d^	H	3-F	H	CN	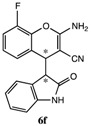	15 h	85%
7	H	4-F	H	CN	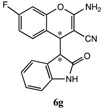	2 h	83%
8	H	5-F	H	CN	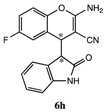	10 min	91%
9	5-F	H	H	CN	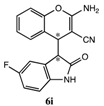	9 min	89%
10	H	H	-ph	CN	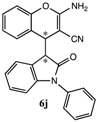	5 min	90%
11	H	H	-CH_3_	CN	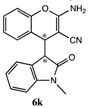	10 min	87%
12 ^e^	H	H	H	-COOEt	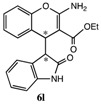	3h	67%
13 ^f^	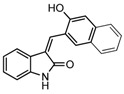				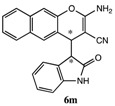	70 min	70%

^a^ All reactions were performed using **4** (0.1 mmol), **5** (0.1 mmol) in THF (0.5 mL). The products were characterized by ^1^H-NMR, ^13^C-NMR and MS. ^b^ Isolated yield after silica chromatography. ^c^ Diastereoisomer ratios (*syn/anti* = 1:1) calculated from the isolated isomers. ^d^ This reaction was performed in room temperature. ^e^ dr = 10:1. ^f^ Single isomer was obtained.

The structures of compounds **6a**–**m** were confirmed by ^1^H-NMR, ^13^C-NMR and MS applied to all the diastereoisomers. In the ^1^H-NMR spectrum of compound **6a**, the two key adjacent hydrogens were observed as two doublets at δ 3.67 ppm and 4.32 ppm. In the ^13^C-NMR spectrum, the characteristic meso methylene carbon resonated at δ 34.3 ppm (C-1) and 49.7 ppm (C-2), providing further evidence for the formation of the product. The NMR spectra of the other compounds **6b**–**m** were consistent with previous reports.

To explore the scope and limitations of this reaction, we further extended the substrates to a variety of other substituents and heterocycles for the preparation of structurally diverse and functionalized 4-substituted-4*H*-chromenes. When Knoevenagel adducts derived from pyrazolone, nitromethane, *N,N*-dimethylbarbituric acid or indanedione were employed, we were pleased to find that the reactions proceeded smoothly in THF (0.5 mL) at 60 °C in 10 min to provide products **6n**–**q** in good yield (75%–90%) (Table 3).

**Table 3:** Synthesis of more substituted 4*H*-chromenes **6n**–**q** ^a,b^.

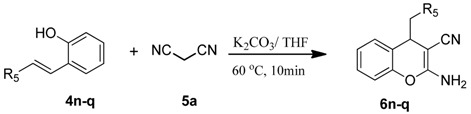

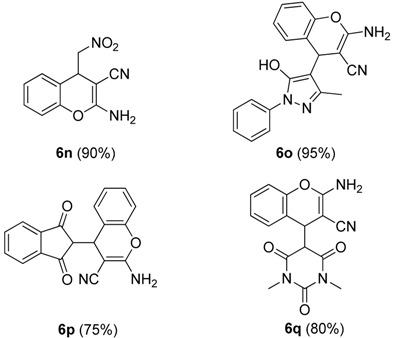

^a^ All reactions were performed using **4** (0.1 mmol), **5** (0.1 mmol) in THF (0.5 mL) at 60 °C in 5, 12, 40, 10 min, respectively. The products were characterized by ^1^H-NMR, ^13^C-NMR and MS. ^b^ Isolated yield after silica gel chromatography.

## 3. Experimental Section

### 3.1. General Information

All chemicals were obtained from commercial sources and used without further purification. Column chromatography was carried out on silica gel (300–400 mesh, Qingdao Marine Chemical Ltd., Qingdao, China). Thin layer chromatography (TLC) was performed on TLC silica gel 60 F254 plates.^1^H-NMR spectra were recorded on a Bruker AVII-400 or AVII-600 MHz NMR spectrometer. The chemical shifts were recorded in ppm relative to tetramethylsilane and with the solvent resonance as the internal standard. Data were reported as follows: chemical shift, multiplicity (s = singlet, d = doublet, t = triplet, q = quartlet, m = multiplet), coupling constants (Hz), integration. ^13^C-NMR data were collected at 100 MHz with complete proton decoupling. Chemical shifts were reported in ppm from the tetramethylsilane with the solvent resonance as internal standard. MS spectra were obtained on a Waters Quattro Premier XETM triple quadrupole mass spectrometer and methanol was used to dissolve the sample. Melting points were recorded on a SGW X-4 melting point instrument (Shanghai Precision & Scientific Instrument Co., Ltd, Shanghai, China).

### 3.2. Experimental Procedures

A mixture of 1,3-dihydro-3-[(2-hydroxyphenyl)methylene]-2*H*-indol-2-one (**4a**, 0.1 mmol), malononitrile (**5a**, 0.1 mmol) and K_2_CO_3_ (0.1 mmol) was stirred in THF (0.5 mL) for 10 min at 60 °C. After completion of the reaction (TLC), the solvent was removed under vacuum. The crude product was subjected to column chromatography on silica gel using petroleum ether/ethyl acetate = 1:1 as the eluent to give **6a**. Compounds **6b**–**q** were synthesized by a similar procedure as described for compound **6a**. For the separation of these compounds, the silica gel column chromatography eluent consisted of appropriate mixtures of petroleum ether and ethyl acetate.

*2-Amino-4-(2-oxoindolin-3-yl)-4H-chromene-3-carbonitrile* (**6a**). Isomer **6aa**: White solid; m.p. 192–194 °C; ^1^H-NMR (400 MHz, TMS, DMSO): δ 3.67 (d, *J* = 2.8 Hz, 1H), 4.32 (d, *J* = 2.8 Hz, 1H), 6.45 (d, *J* = 7.2 Hz, 1H), 6.71 (s, 2H), 6.76–6.81 (m, 2H), 7.02 (d, *J* = 8.0 Hz, 1H), 7.11–7.16 (m, 2H), 7.23–7.25 (m, 1H), 7.30–7.34 (m, 1H), 10.41 (s, 1H); ^13^C-NMR (100 MHz, DMSO): δ 36.6, 50.5, 53.1, 109.2, 115.8, 120.0, 120.9, 121.5, 123.5, 124.6, 126.8, 128.0, 128.2, 128.6, 143.5, 149.7, 162.1, 176.0; MS: *m/z* = 326 [M+Na]^+^. Isomer **6ab**: White solid; m.p. 191–193 °C; ^1^H-NMR (400 MHz, TMS, DMSO): δ 3.63 (d, *J =* 2.8 Hz, 1H), 4.25 (d, *J =* 2.8 Hz, 1H), 6.61 (d, *J =* 8.0 Hz, 1H), 6.78 (d, *J =* 8.0 Hz, 1H), 6.84 (t, *J =* 8.0 Hz, 1H), 6.98–7.07 (m, 2H), 7.09–7.19 (m, 5H), 10.43 (s, 1H); ^13^C-NMR (100 MHz, DMSO): δ 37.2, 52.4, 52.7, 109.1, 115.6, 119.7, 120.2, 121.1, 123.6, 124.2, 126.6, 127.6, 128.0, 128.4, 142.9, 149.3, 161.9, 176.7; MS: *m/z* = 326 [M+Na]^+^.

*2-Amino-6-methyl-4-(2-oxoindolin-3-yl)-4H-chromene-3-carbonitrile* (**6b**). Isomer **6ba**: White solid; m.p. 209–211 °C; ^1^H-NMR (400 MHz, TMS, DMSO): δ 2.16 (s, 3H), 3.62 (d, *J =* 3.2 Hz, 1H), 4.19 (d, *J =* 3.2 Hz, 1H), 6.62 (d, *J* = 7.6 Hz, 1H), 6.67 (d, *J* = 8.0 Hz, 1H), 6.82–6.86 (m, 1H), 6.90–6.93 (m, 1H), 6.96 (s, 1H), 7.03–7.07 (m, 3H), 7.17 (d, *J* = 7.6 Hz, 1H), 10.45 (s, 1H); ^13^C-NMR (100 MHz, DMSO): δ 20.3, 37.2, 52.2, 52.7, 109.1, 115.3, 119.4 120.3, 121.1, 123.7, 126.7, 127.8, 128.0, 128.9, 133.0, 143.0, 147.3, 162.1, 176.7; MS: *m/z* = 340 [M+Na]^+^. Isomer **6bb**: White solid; m.p. 136–139 °C; ^1^H-NMR (400 MHz, TMS, CDCl_3_): δ 2.29 (s, 3H), 3.68 (d, *J =* 3.2 Hz, 1H), 4.43 (d, *J =* 3.2 Hz, 1H), 4.65 (s, 2H), 6.46 (d, *J =* 7.2 Hz, 1H), 6.83–6.89 (m, 3H), 6.98 (s, 1H), 7.06–7.08 (m, 1H), 7.16–7.20 (m, 1H), 8.79 (s, 1H); ^13^C-NMR (100 MHz, CDCl_3_): δ 20.8, 37.4, 53.5, 53.7, 110.1, 116.1, 120.1, 120.6, 122.0, 124.2, 126.4, 128.3, 128.5, 129.4, 135.0, 142.6, 147.9, 162.1, 177.4; MS: *m/z* = 340 [M+Na]^+^.

*2-Amino-6-chloro-4-(2-oxoindolin-3-yl)-4H-chromene-3-carbonitrile* (**6c**). Isomer **6ca**: White solid; m.p. 183–184 °C; ^1^H-NMR (400 MHz, TMS, DMSO): δ 3.67 (d, *J =* 3.2 Hz, 1H), 4.26 (d, *J =* 3.2 Hz, 1H), 6.65 (d, *J =* 8.0 Hz, 1H), 6.83–6.89 (m, 2H), 7.07–7.23 (m, 6H), 10.53 (s, 1H); ^13^C-NMR (100 MHz, DMSO): δ 37.5, 52.1, 53.2, 109.7, 118.0, 120.3, 121.8, 122.6, 124.4, 126.9, 127.8, 128.1, 128.7, 128.8, 143.4, 148.8, 162.3, 177.1; MS: *m/z* = 360 [M+Na]^+^. Isomer **6cb**: White solid; m.p. 170–172 °C; ^1^H-NMR (400 MHz, TMS, DMSO): δ 3.72 (d, *J =* 3.2 Hz, 1H), 4.33 (d, *J =* 3.2 Hz, 1H), 6.55 (d, *J =* 8.0 Hz, 1H), 6.76–6.85 (m, 4H), 7.05 (d, *J =* 8.4 Hz, 1H), 7.16 (t, *J =* 8.0 Hz, 1H), 7.24–7.25 (m, 1H), 7.34–7.37 (m, 1H), 10.40 (s, 1H); ^13^C-NMR (100 MHz, DMSO): δ 37.0, 50.6, 53.2, 109.7, 118.2, 120.4, 121.5, 123.9, 124.1, 127.1, 128.4, 128.6, 128.7, 129.0, 144.0, 149.0, 162.4, 176.4; MS: *m/z* = 360 [M+Na]^+^.

*2-Amino-4-(6-chloro-2-oxoindolin-3-yl)-4H-chromene-3-carbonitrile* (**6d**). Isomer **6da**: White solid; m.p. 184–186 °C; ^1^H-NMR (400 MHz, TMS, DMSO): δ 3.67 (d, *J =* 3.2 Hz, 1H), 4.26 (d, *J =* 3.2 Hz, 1H), 6.63–6.64 (m, 1H), 6.83 (d, *J =* 8.0 Hz, 1H), 6.93 (dd, *J =* 8.0 Hz, *J =* 2.0 Hz, 1H), 7.01–7.20 (m, 6H), 10.57 (s, 1H); ^13^C-NMR (100 MHz, DMSO): δ 37.2, 51.9, 52.5, 109.1, 115.7, 119.7, 120.1, 120.8, 124.3, 125.1, 125.8, 127.6, 128.6, 132.3, 144.5, 149.3, 162.0, 176.6; MS: *m/z* = 360 [M+Na]^+^. Isomer **6db**: White solid; m.p. 179–182 °C; ^1^H-NMR (400 MHz, TMS, DMSO): δ 3.69 (d, *J =* 2.8 Hz, 1H), 4.32 (d, *J =* 2.8 Hz, 1H), 6.40 (d, *J =* 8.0 Hz, 1H), 6.73 (s, 2H), 6.78 (d, *J* = 2.0 Hz, 1H), 6.85 (dd, *J =* 8.0 Hz, *J =* 2.0 Hz, 1H), 7.02 (d, *J* = 8.0 Hz, 1H), 7.16 (t, *J* = 7.6 Hz, 1H), 7.27–7.35 (m, 2H), 10.56 (s, 1H); ^13^C-NMR (100 MHz, DMSO): δ 36.6, 49.9, 52.8, 109.2, 115.9, 120.0, 120.6, 121.2, 124.8, 124.9, 125.8, 128.3, 128.8, 132.4, 145.1, 149.6, 162.1, 176.1; MS: *m/z* = 360 [M+Na]^+^.

*2-Amino-6-bromo-4-(2-oxoindolin-3-yl)-4H-chromene-3-carbonitrile* (**6e**). Isomer **6ea**: White solid; m.p. 118–120 °C; ^1^H-NMR (400 MHz, TMS, DMSO): δ 3.66 (d, *J =* 2.8 Hz, 1H), 4.25 (d, *J =* 2.8 Hz, 1H), 6.65 (d, *J =* 8.0 Hz, 1H), 6.78 (t, *J =* 8.0 Hz, 1H), 6.87 (t, *J =* 8.0 Hz, 1H), 7.08 (t, *J =* 8.0 Hz, 1H), 7.14 (s, 2H), 7.20 (d, *J =* 8.0 Hz, 1H), 7.30 (dd, *J =* 8.0 Hz, *J =* 2.0 Hz, 1H), 7.37 (d, *J =* 2.0 Hz, 1H), 10.53 (s, 1H); ^13^C-NMR (100 MHz, DMSO): δ 36.9, 51.7, 52.7, 109.2, 115.6, 117.9, 119.8, 121.3, 122.6, 123.9, 126.4, 128.2, 130.2, 131.2, 142.9, 148.8, 161.7, 176.5; MS: *m/z* = 382 [M+H]^+^. Isomer **6eb**: White solid; m.p. 172–175 °C; ^1^H-NMR (400 MHz, TMS, DMSO): δ 3.71 (d, *J =* 3.2 Hz, 1H), 4.33 (d, *J =* 3.2 Hz, 1H), 6.57 (d, *J =*7.6 Hz, 1H), 6.76–6.85 (m, 4H), 6.98 (d, *J =* 8.0 Hz, 1H), 7.16 (t, *J =* 8.0 Hz, 1H), 7.34–7.35 (m, 1H), 7.47 (dd, *J =* 8.0 Hz, *J =* 2.0 Hz, 1H), 10.39 (s, 1H); ^13^C-NMR (100 MHz, DMSO): δ 36.4, 50.2, 52.7, 109.2, 116.0, 118.0, 119.9, 121.1, 123.6, 123.8, 126.6, 128.2, 130.8, 131.4, 143.5, 148.9, 161.8, 175.9; MS: *m/z* = 380 [M−H]^−^.

*2-Amino-8-fluoro-4-(2-oxoindolin-3-yl)-4H-chromene-3-carbonitrile* (**6f**). Isomer **6fa**: White solid; m.p. 197–199 °C; ^1^H-NMR (400 MHz, TMS, DMSO): δ 3.66 (d, *J =* 3.2 Hz, 1H), 4.29 (d, *J =* 3.2 Hz, 1H), 4.63 (d, *J =* 8.0 Hz, 1H), 6.87 (t, *J =* 7.2 Hz, 1H), 6.97–7.11 (m, 4H), 7.19–7.24 (m, 3H), 10.43 (s, 1H); ^13^C-NMR (100 MHz, DMSO): δ 37.0, 52.3, 52.7, 109.1, 115.1(d, *J* = 7 Hz), 119.7, 121.2, 122.7, 122.9 (d, *J* = 3 Hz), 123.7, 124.2 (d, *J* = 7 Hz), 126.4, 128.2, 137.5 (d, *J* = 11 Hz), 143.0, 149.1(d, *J* = 245 Hz), 161.3, 176.5; MS: *m/z* = 344 [M+Na]^+^. Isomer **6fb**: White solid; m.p. 133–135 °C; ^1^H-NMR (400 MHz, TMS, DMSO): δ 3.68 (d, *J =* 2.8 Hz, 1H), 4.36 (d, *J =* 2.8 Hz, 1H), 4.56 (d, *J =* 7.2 Hz, 1H), 6.77 (d, *J =* 7.6 Hz, 1H), 6.82–6.89 (m, 3H), 6.98 (d, *J =* 8.0 Hz, 1H), 7.09–7.18 (m, 2H), 7.24–7.29 (m, 1H), 10.39 (s, 1H); ^13^C-NMR (100 MHz, DMSO): δ 36.5, 50.6, 52.8, 109.3, 115.2(d, *J* = 17Hz), 119.8, 121.1, 123.4 (d, *J* = 3 Hz), 123.5, 124.0, 124.5(d, *J* = 7 Hz), 126.6, 128.2, 137.8 (d, *J* = 11Hz), 143.5, 149.3 (d, *J* = 245Hz), 161.4, 175.9; MS: *m/z* = 344 [M+Na]^+^.

*2-Amino-7-fluoro-4-(2-oxoindolin-3-yl)-4H-chromene-3-carbonitrile* (**6g**). Isomer **6ga**: White solid; m.p. 167–169 °C; ^1^H-NMR (400 MHz, TMS, DMSO): δ 3.62 (d, *J =* 2.8 Hz, 1H), 4.23 (d, *J =* 2.8 Hz, 1H), 6.63 (d, *J =* 7.6 Hz, 1H), 6.68–6.71 (m, 1H), 6.84–6.93 (m, 2H), 7.07 (t, *J =* 8.0 Hz, 1H), 7.16–7.20 (m, 4H), 10.44 (s, 1H); ^13^C-NMR (100 MHz, DMSO): δ 36.8, 52.4, 52.7, 103.1 (d, *J* = 25 Hz), 109.1, 111.3 (d, *J* = 22 Hz), 116.1, 119.9, 121.2, 123.7, 126.5, 128.1, 129.1(d, *J* = 10 Hz), 142.9, 150.0 (d, *J* = 12 Hz), 161.1 (d, *J* = 243 Hz), 161.5, 176.6; MS: *m/z* = 344 [M+Na]^+^. Isomer **6gb**: White solid; m.p. 165–169 °C; ^1^H-NMR (400 MHz, TMS, DMSO): δ3.66 (d, *J =* 2.8 Hz, 1H), 4.31 (d, *J =* 2.8 Hz, 1H), 6.54 (d, *J =* 7.6 Hz, 1H), 6.68–6.71 (m, 1H), 6.75–6.86 (m, 3H), 6.89–6.94 (m, 1H), 6.96–7.02 (m, 1H), 7.04–7.10 (m, 1H), 7.13–7.26 (m, 1H), 10.38 (s, 1H); ^13^C-NMR (100 MHz, DMSO): δ 36.1, 50.5, 52.9, 103.3 (d, *J* = 25 Hz), 109.2, 111.7 (d, *J* = 22 Hz), 119.9, 121.1, 123.6, 126.7, 128.1, 129.4 (d, *J* = 10 Hz), 129.8 (d, *J* = 10 Hz), 143.5, 150.2 (d, *J* = 12 Hz), 161.4 (d, *J* = 245 Hz), 161.7, 176.0; MS: *m/z* = 344 [M+Na]^+^.

*2-Amino-6-fluoro-4-(2-oxoindolin-3-yl)-4H-chromene-3-carbonitrile* (**6h**). Isomer **6ha**: White solid; m.p. 212–217 °C; ^1^H-NMR (400 MHz, TMS, DMSO): δ 3.66 (d, *J =* 2.8 Hz, 1H), 4.25 (d, *J =* 2.8 Hz, 1H), 6.64 (d, *J =* 7.6 Hz, 1H), 6.82–6.88 (m, 2H), 6.93–7.01 (m, 2H), 7.05–7.11 (m, 3H), 7.18–7.20 (m, 1H), 10.49 (s, 1H); ^13^C-NMR (100 MHz, DMSO): δ 37.3, 51.5, 52.6, 109.1, 113.7 (d, *J* = 24 Hz), 115.2 (d, *J* = 24 Hz), 117.3 (d, *J* = 8 Hz), 120.0, 121.3, 121.7 (d, *J* = 8 Hz), 123.8, 126.4, 128.1, 142.9, 145.7, 157.8 (d, *J* = 238 Hz), 161.9, 176.6; MS: *m/z* = 344 [M+Na]^+^. Isomer **6hb**: White solid; m.p. 202–203 °C; ^1^H-NMR (400 MHz, TMS, DMSO): δ3.72 (d, *J =* 2.8 Hz, 1H), 4.33 (d, *J =* 2.8 Hz, 1H), 6.49 (d, *J =* 7.6 Hz, 1H), 6.72 (s, 2H), 6.76–6.83 (m, 2H), 7.04–7.10 (m, 2H), 7.14–7.18 (m, 2H), 10.40 (s, 1H); ^13^C-NMR (100 MHz, DMSO): δ36.7, 49.7, 52.7, 109.3, 114.4 (d, *J* = 24 Hz), 115.5 (d, *J* = 24 Hz), 117.4, 117.5, 119.9, 121.0, 123.2, 123.3, 123.5, 126.6, 128.1, 144.8 (d, *J* = 249 Hz), 162.1, 175.9; MS: *m/z* = 344 [M+Na]^+^.

*2-Amino-4-(5-fluoro-2-oxoindolin-3-yl)-4H-chromene-3-carbonitrile* (**6i**). Isomer **6ia**: White solid; m.p. 183–185 °C; ^1^H-NMR (400 MHz, TMS, DMSO): δ 3.68 (d, *J =* 2.8 Hz, 1H), 4.26 (d, *J =* 2.8 Hz, 1H), 6.58–6.61 (m, 1H), 6.82–6.84 (m, 1H), 6.87–6.93 (m, 1H), 6.99–7.05 (m, 2H), 7.12–7.19 (m, 4H), 10.43 (s, 1H); ^13^C-NMR (100 MHz, DMSO): δ 37.1, 50.7.6, 54.0, 110.2, 110.3, 111.7.0 (d, *J* = 25 Hz), 114.8 (d, *J* = 23 Hz), 116.1, 120.5, 121.6, 124.8, 128.2, 128.8, 140.3, 150.2, 157.9 (d, *J* = 234 Hz), 162.7, 176.4; MS: *m/z* = 344 [M+Na]^+^. Isomer **6ib**: White solid; m.p. 130–134 °C; ^1^H-NMR (400 MHz, TMS, DMSO): δ 3.71 (d, *J =* 2.8 Hz, 1H), 4.34 (d, *J =* 2.8 Hz, 1H), 6.73–6.77 (m, 3H), 6.96–7.05 (m, 2H), 7.12–7.17 (m, 2H), 7.24–7.25 (m, 1H), 7.32–7.36 (m, 1H), 10.43 (s, 1H); ^13^C-NMR (100 MHz, DMSO): δ37.1, 50.7, 54.0, 110.2, 110.3, 111.7 (d, *J* = 24 Hz), 114.8 (d, *J* = 24 Hz), 116.3, 120.5, 121.6, 125.3, 128.8, 129.3, 140.3, 150.2, 157.8 (d, *J* = 235 Hz), 162.7, 176.4; MS: *m/z* = 344 [M+Na]^+^.

*2-Amino-4-(2-oxo-1-phenylindolin-3-yl)-4H-chromene-3-carbonitrile* (**6j**). Isomer **6ja**: White solid; m.p. 186–187 °C; ^1^H-NMR (400 MHz, TMS, CDCl_3_): δ 3.95 (d, *J =* 3.2 Hz, 1H), 4.55 (d, *J =* 3.2 Hz, 1H), 4.92 (s, 2H), 6.50 (d, *J =* 8.0 Hz, 1H), 6.74 (t, *J =* 8.0 Hz, 1H), 6.97–7.00 (m, 2H), 7.05–7.12 (m, 2H), 7.20–7.24 (m, 3H), 7.36 (d, *J =* 8.0Hz, 1H), 7.40 (d, *J =* 7.6 Hz, 1H), 7.47–7.51 (m, 2H); ^13^C-NMR (100 MHz, DMSO): δ 29.7, 38.2, 52.6, 109.1, 116.0, 118.8, 119.4, 122.9, 124.4, 124.6, 125.5, 126.5, 128.1, 128.2, 128.3, 128.7, 129.6, 134.2, 144.6, 149.5, 161.6, 174.8; MS: *m/z* = 402 [M+Na]^+^. Isomer **6jb**: White solid; m.p. 192–194 °C; ^1^H-NMR (400 MHz, TMS, DMSO): δ 3.97 (d, *J* = 2.8 Hz, 1H), 4.45 (d, *J* = 2.8 Hz, 1H), 6.55 (d, *J* = 7.6 Hz, 1H), 6.65 (d, *J* = 7.6 Hz, 1H), 6.79 (s, 2H), 6.92 (t, *J* = 7.2 Hz, 1H), 7.06 (d, *J* = 7.6 Hz, 1H), 7.19 (d, *J* = 7.6 Hz, 2H), 7.30 (d, *J* = 7.2 Hz, 1H), 7.35–7.40 (m, 3H), 7.45 (d, *J* = 7.6 Hz, 1H), 7.55 (d, *J* = 7.6 Hz, 2H) ; MS: *m/z* = 402 [M+Na]^+^.

*2-Amino-4-(1-methyl-2-oxoindolin-3-yl)-4H-chromene-3-carbonitrile* (**6k**). Isomer **6ka**: White solid; m.p. 208–211 °C; ^1^H-NMR (400 MHz, TMS, DMSO): δ 3.02 (s, 3H), 3.68 (d, *J =* 3.2 Hz, 1H), 4.26 (d, *J =* 3.2 Hz, 1H), 6.73–6.76 (m, 2H), 6.90–7.02 (m, 3H), 7.06–7.16 (m, 4H), 7.21 (d, *J =* 7.2Hz, 1H); ^13^C-NMR (100 MHz, DMSO): δ 25.8, 37.7, 52.1, 52.3, 108.0, 115.6, 119.2, 120.2, 121.8, 123.4, 124.0, 125.7, 127.2, 128.1, 128.5, 144.4, 149.2, 161.9, 174.7; MS: *m/z* = 340 [M+Na]^+^. Isomer **6kb**: White solid; m.p. 202–205 °C; ^1^H-NMR (400 MHz, TMS, DMSO): δ 3.07 (s, 3H), 3.75 (d, *J =* 2.8 Hz, 1H), 4.34 (d, *J =* 2.8 Hz, 1H), 6.35 (d, *J =* 7.2 Hz, 1H), 6.64 (s, 2H), 6.85 (t, *J =* 7.2 Hz, 1H), 6.96 (d, *J =* 7.6 Hz, 1H), 7.03 (d, *J =* 8.0 Hz, 1H), 7.16–7.22 (m, 1H), 7.25 (t, *J =* 8.0 Hz, 1H), 7.32–7.39 (m, 2H); ^13^C-NMR (100 MHz, DMSO): δ 26.0, 37.0, 49.5, 52.6, 108.2, 115.8, 119.7, 121.2, 121.6, 123.1, 124.8, 125.9, 128.2, 128.4, 128.7, 145.0, 149.5, 161.9, 174.3; MS: *m/z* = 340 [M+Na]^+^.

*ethyl 2-Amino-4-(2-oxoindolin-3-yl)-4H-chromene-3-carboxylate* (**6l**). White solid; m.p. 93–96 °C; ^1^H-NMR (400 MHz, TMS, DMSO): δ1.38 (t, *J =* 7.2 Hz, 3H), 3.87 (d, *J =* 3.2 Hz, 1H), 4.30–4.36 (m, 2H), 4.77 (d, *J =* 3.2 Hz, 1H), 6.60–6.66 (m, 2H), 6.84–6.90 (m, 2H), 6.97–7.06 (m, 2H), 7.23–7.28 (m, 2H), 8.32 (s, 1H); ^13^C-NMR (100 MHz, DMSO): δ 14.7, 36.7, 53.2, 59.8, 75.5, 109.1, 115.3, 121.4 122.1, 124.1, 124.6, 127.3, 127.8, 127.9, 128.4, 141.6, 149.9, 162.1, 169.1, 178.7; MS: *m/z* = 373 [M+Na]^+^.

*2-Amino-4-(2-oxoindolin-3-yl)-4H-benzo[g]chromene-3-carbonitrile* (**6m**). White solid; m.p. 201–205 °C; ^1^H-NMR (400 MHz, TMS, DMSO): δ 3.69 (d, *J =* 2.8 Hz, 1H), 4.90 (d, *J =* 2.8 Hz, 1H), 6.24 (d, *J =* 7.2 Hz, 1H), 6.72–6.76 (m, 3H), 6.82 (d, *J =* 7.6 Hz, 1H), 7.11–7.15 (m, 1H), 7.30 (d, *J =* 9.2 Hz, 1H ), 7.55–7.59 (m, 1H), 7.71–7.75 (m, 1H), 8.00–8.05 (m, 2H), 8.13 (d, *J =* 8.4 Hz, 1H), 10.62 (s, 1H); ^13^C-NMR (100 MHz, DMSO): δ 34.3, 49.7, 51.5, 109.4, 114.6, 116.6, 119.9, 121.0, 122.0, 123.4, 125.3, 126.5, 127.9, 128.0, 129.1, 129.2, 129.8, 130.9, 143.8, 147.6, 162.1, 176.5; MS: *m/z* = 376 [M+Na]^+^.

*2-Amino-4-(nitromethyl)-4H-chromene-3-carbonitrile* (**6n**). White solid; m.p. 144–146 °C; ^1^H-NMR (400 MHz, TMS, DMSO): δ 4.32 (t, *J =* 5.2 Hz, 1H), 4.67 (dd, *J =* 12.0 Hz, *J =*5.2 Hz, 1H), 4.80 (dd, *J =* 12.0 Hz, *J =*5.2 Hz, 1H), 7.04 (d, *J* = 8.4 Hz, 1H), 7.17–7.21 (m, 3H), 7.31–7.36 (m, 2H); ^13^C-NMR (100 MHz, DMSO): δ 34.6, 49.9, 80.7, 116.1, 119.4, 119.7, 124.7, 128.3, 129.1, 149.4, 162.1; MS: *m/z* = 254 [M+Na]^+^.

*2-Amino-4-(5-hydroxy-3-methyl-1-phenyl-1H-pyrazol-4-yl)-4H-chromene-3-carbonitrile* (**6o**). White solid; m.p. 150–152 °C; ^1^H-NMR (600 MHz, TMS, DMSO): δ 1.83 (s, 3H), 4.93 (s, 1H), 6.65 (s, 2H), 6.94–7.05 (m, 2H), 7.15–7.20 (m, 3H), 7.26–7.29 (m, 4H), 7.88–7.90 (m, 2H); MS: *m/z* = 383 [M+K]^+^.

*2-Amino-4-(1,3-dioxo-2,3-dihydro-1H-inden-2-yl)-4H-chromene-3-carbonitrile* (**6p**). White solid; m.p. 85–88 °C; ^1^H-NMR (400 MHz, TMS, DMSO): δ 3.61 (d, *J =* 2.8 Hz, 1H), 4.35 (d, *J =* 2.8 Hz, 1H), 6.94–7.11 (m, 3H), 7.19–7.23 (m, 1H), 7.87–7.92 (m, 4H); MS: *m/z* = 315 [M−H]^−^.

*2-Amino-4-(1,3-dimethyl-2,4,6-trioxohexahydropyrimidin-5-yl)-4H-chromene-3-carbonitrile* (**6q**). White solid; m.p. 195–198 °C; ^1^H-NMR (400 MHz, TMS, DMSO): δ 3.01 (s, 3H), 3.07 (s, 3H), 3.67 (d, *J =* 2.8 Hz, 1H), 4.37 (d, *J =* 2.8 Hz, 1H), 6.98–7.00 (m, 1H), 7.16–7.20 (m, 2H), 7.30–7.32 (m, 1H); MS: *m/z* = 325 [M−H]^−^.

## 4. Conclusions

In conclusion, we have demonstrated an efficient approach for the synthesis of functionalised 4-substituted-2-amino-3-cyano-4*H*-chromenes via a tandem conjugate addition-cyclization reaction of malononitrile and a range of Knoevenagel adducts using K_2_CO_3 _as catalyst. A range of 4-substituted-2-amino-3-cyano-4*H*-chromenes were thus obtained in moderate to high yields (up to 98%). This synthetic method offers several advantages, including milder reaction conditions, an economical catalyst system, shorter time for completion and simple process, all which make it an efficient route for the synthesis of 2-amino-4*H*-chromenes. Moreover, for 4-indolylchromenes, the two isomers formed could be isolated one step silica gel chromatography. Further study on the antibacterial and antitumor activities of these compounds is underway.

## Supplementary Materials

Supplementary materials can be accessed at: http://www.mdpi.com/1420-3049/19/7/10218/s1.
