# Supplementary material for: K2CO3-Mediated Synthesis of Functionalised 4-Substituted-2-amino-3-cyano-4H-chromenes via Michael-Cyclization Reactions
Source: Molecules. 2014 Nov 25;19(12):19253–68. doi: 10.3390/molecules191219253 (PMC6270755; doi:10.3390/molecules191219253)

Supplementary

Copies of NMR Spectra for 4-substituted-2-amino-3-cyano-4*H*-chromenes.

Figure S1. H-NMR of 6aa.

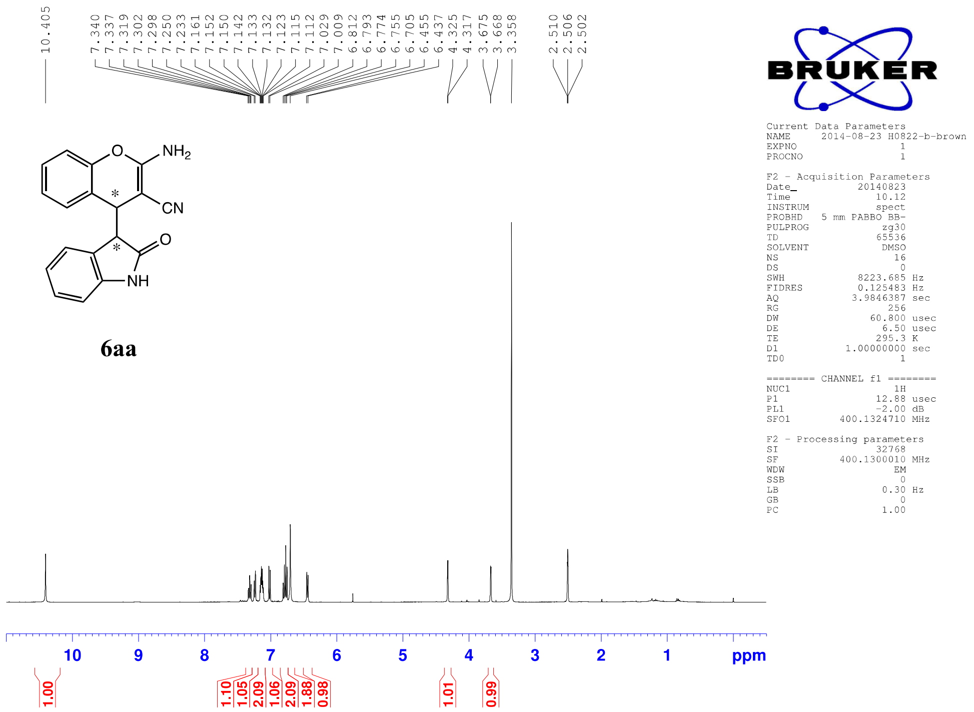

Figure S2. C-NMR of 6aa.

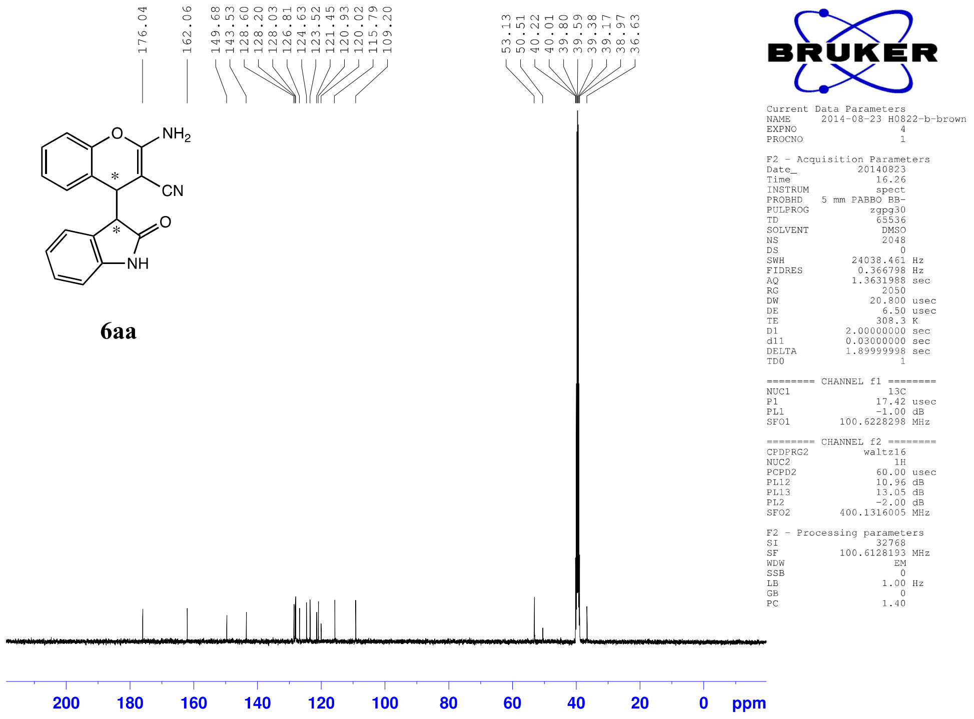

Figure S3. H-NMR of 6ab.

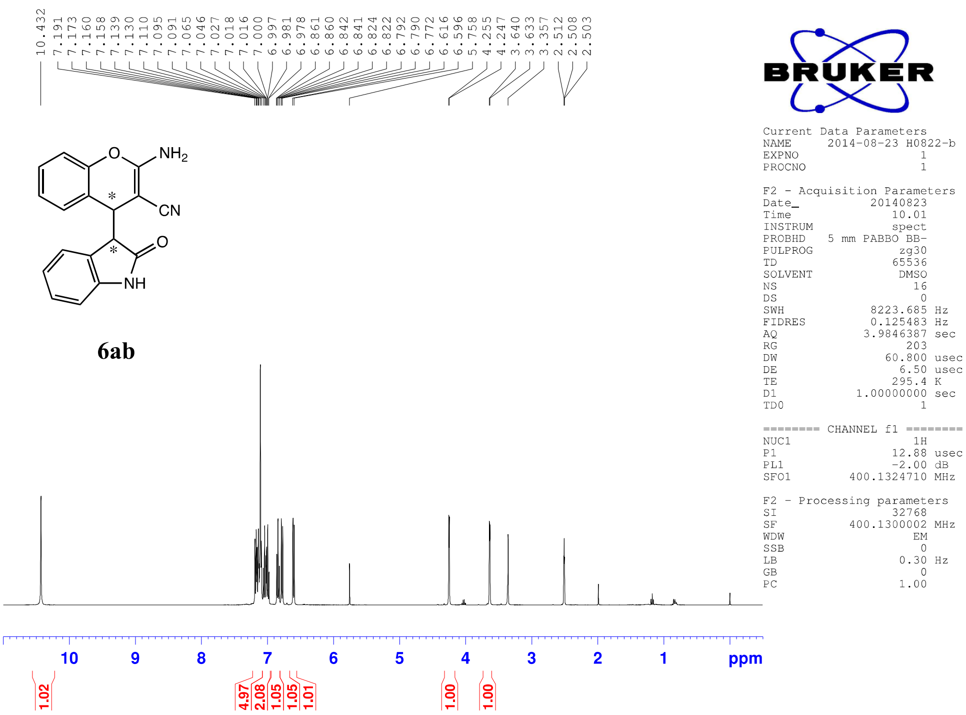

Figure S4. C-NMR of 6ab.

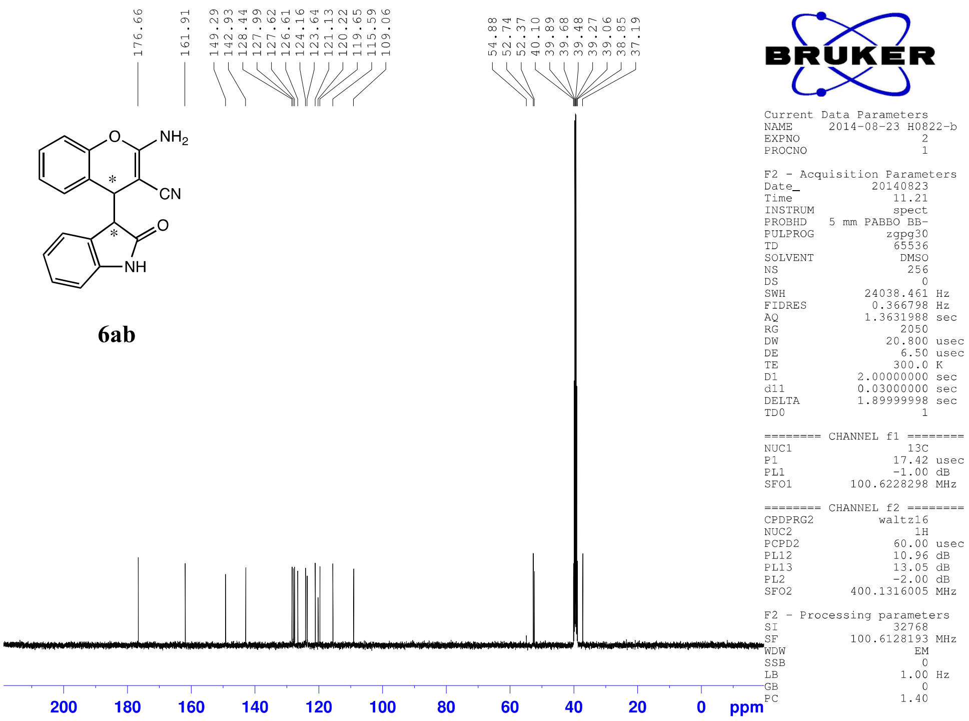

Figure S5. H-NMR of 6ba.

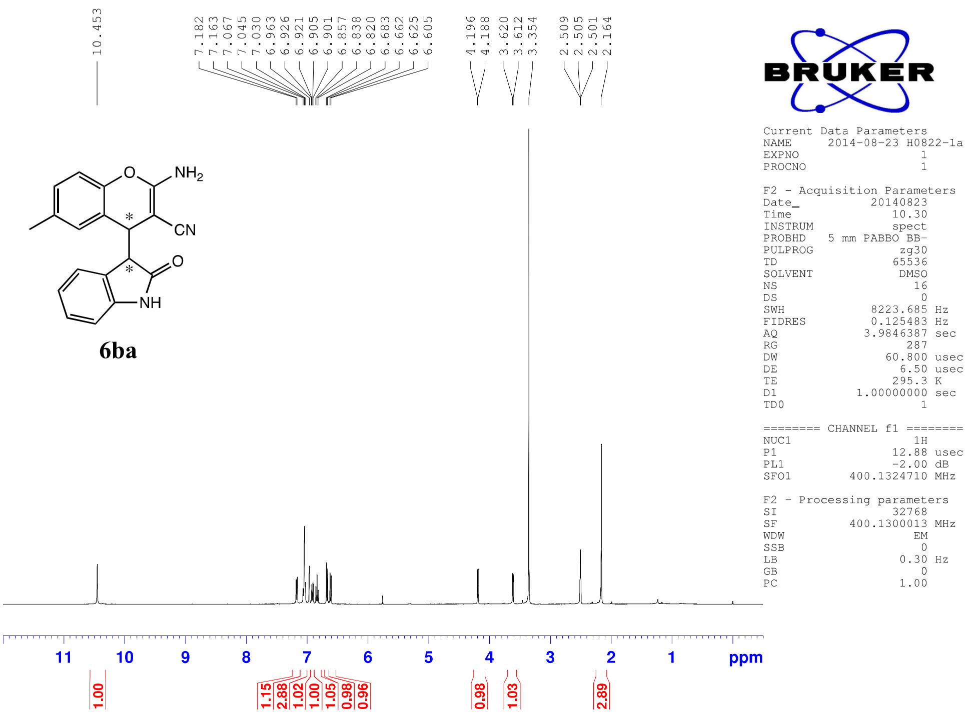

Figure S6. C-NMR of 6ba.

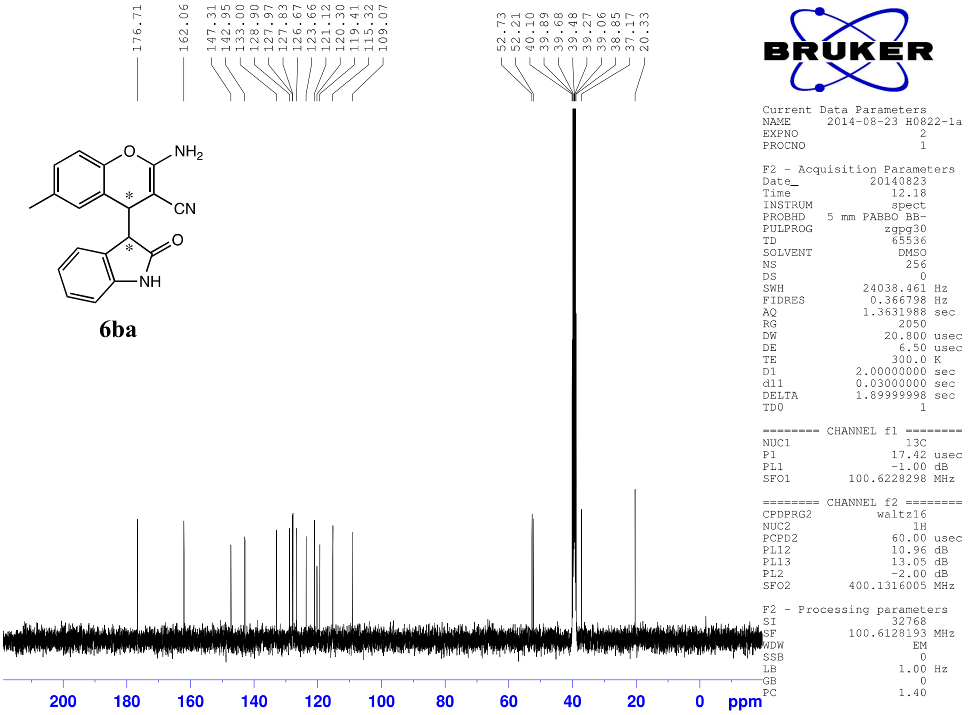

Figure S7. H-NMR of 6bb.

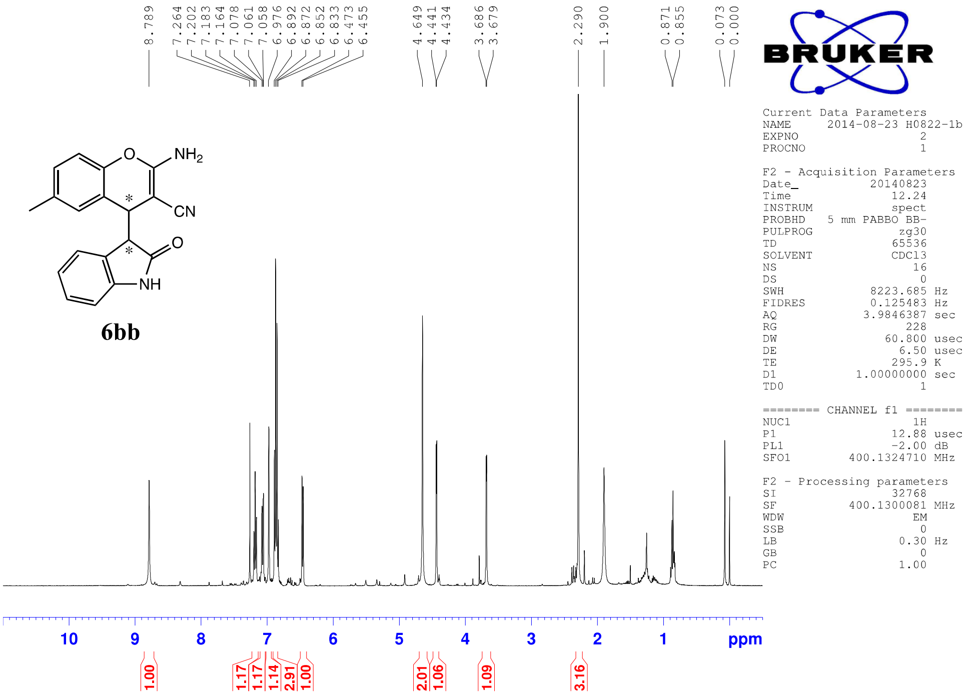

Figure S8. C-NMR of 6bb.

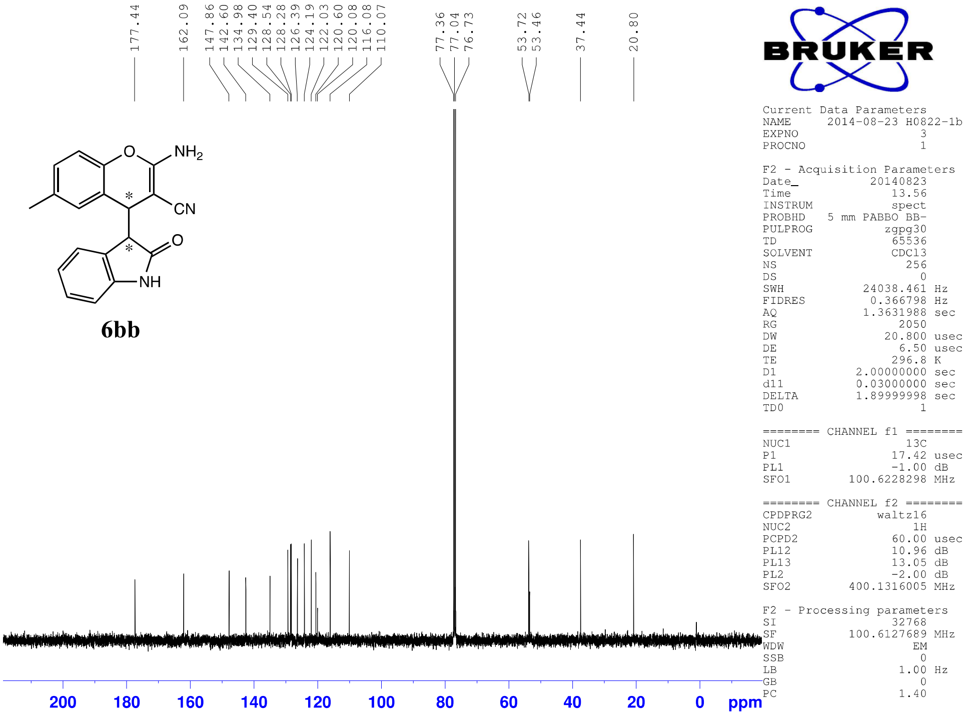

Figure S9. H-NMR of 6ca.

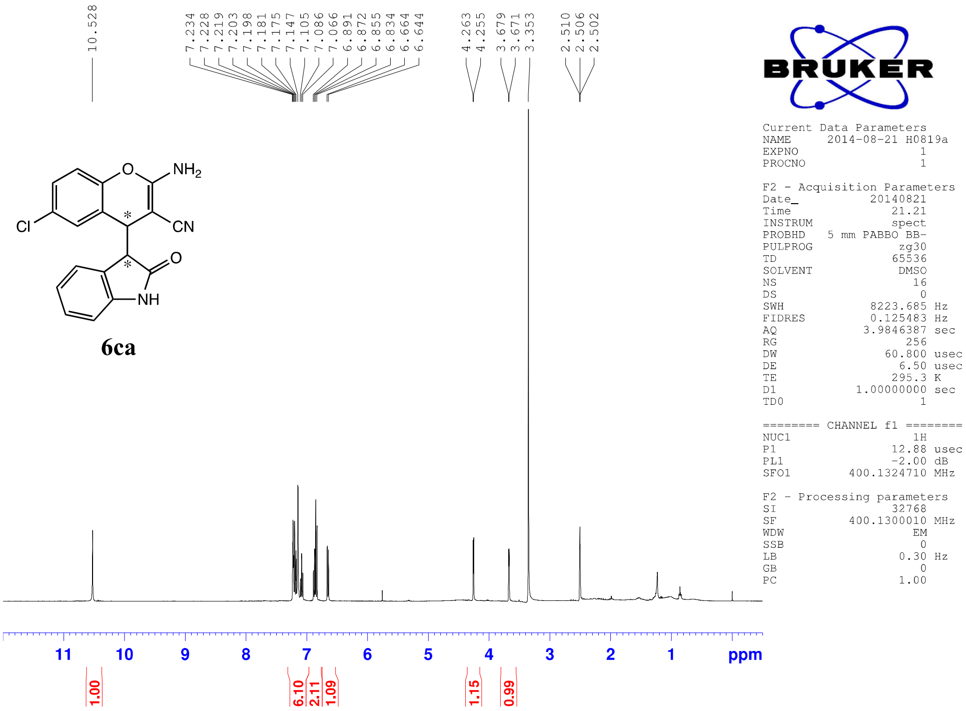

Figure S10. H-NMR of 6ca.

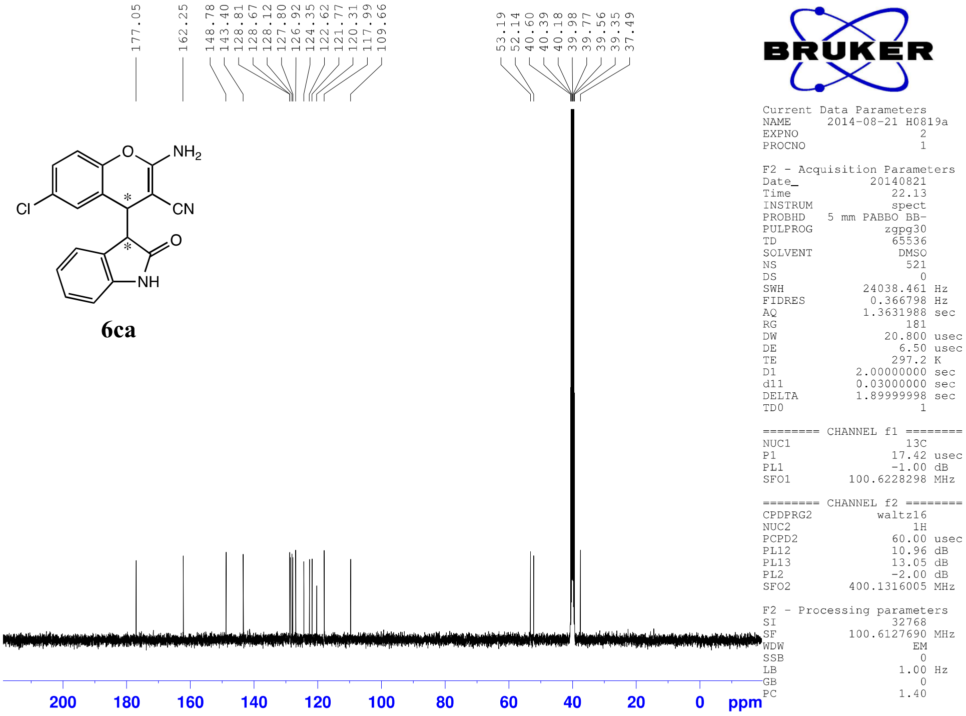

Figure S11. H-NMR of 6cb.

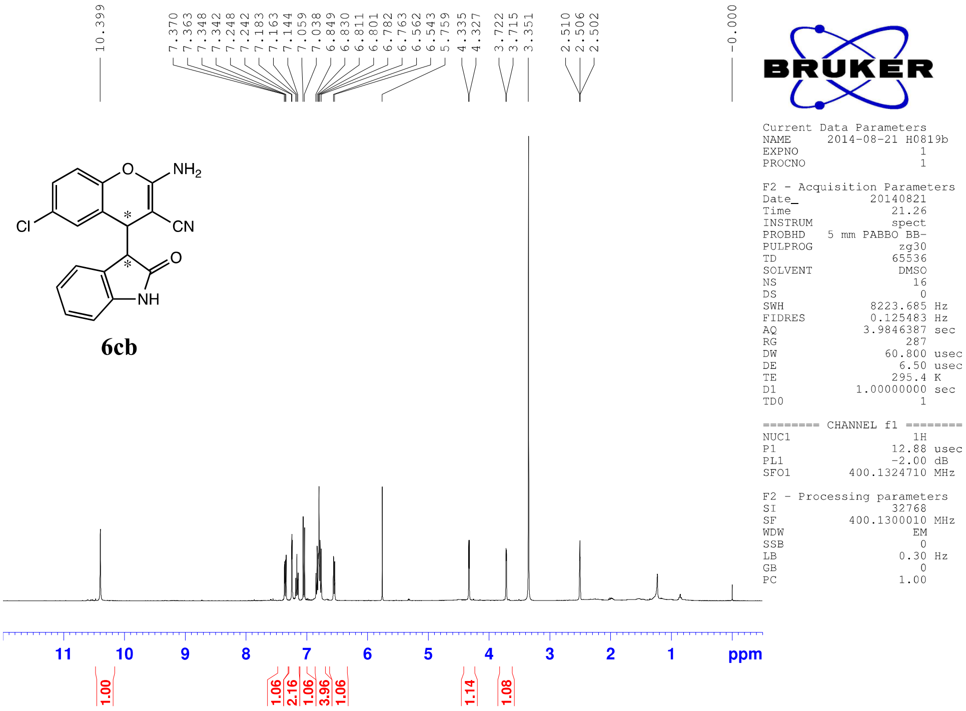

Figure S12. C-NMR of 6cb.

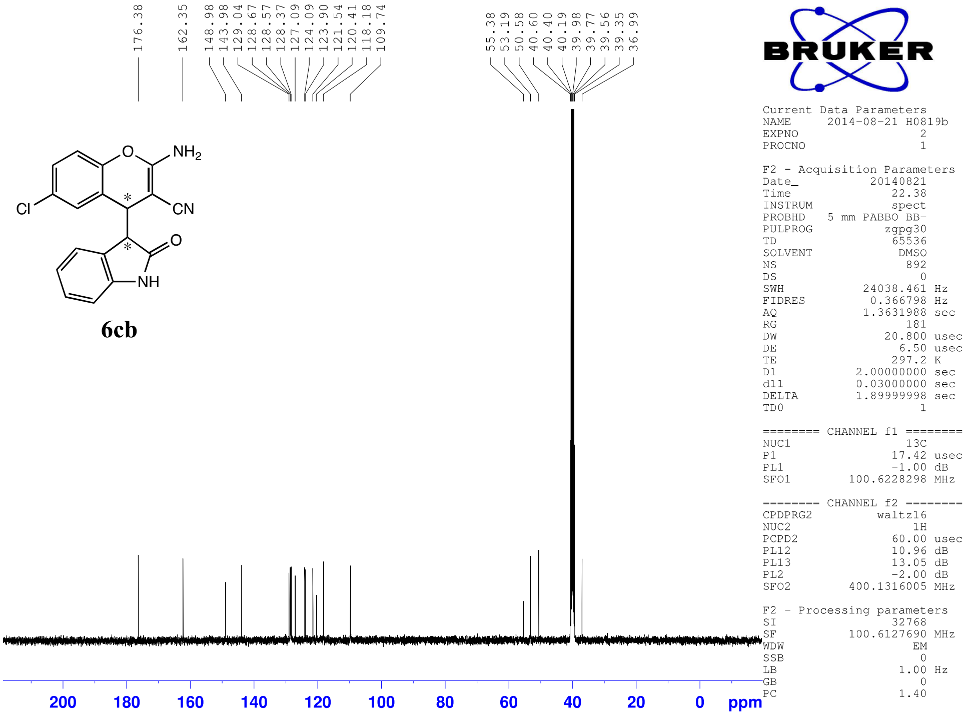

Figure S13. H-NMR of 6da.

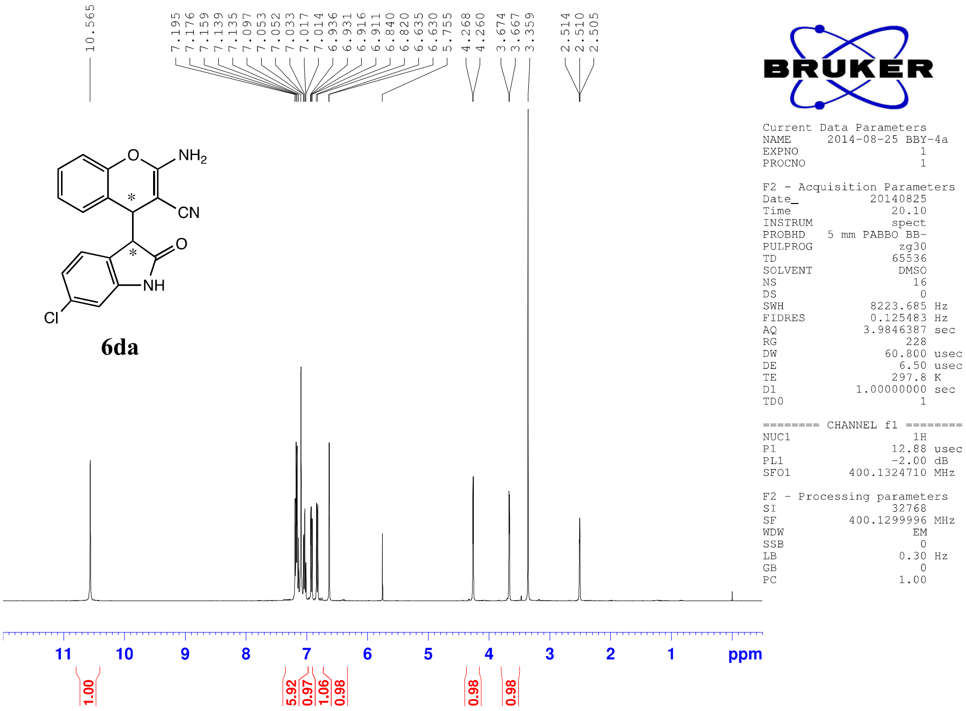

Figure S14. C-NMR of 6da.

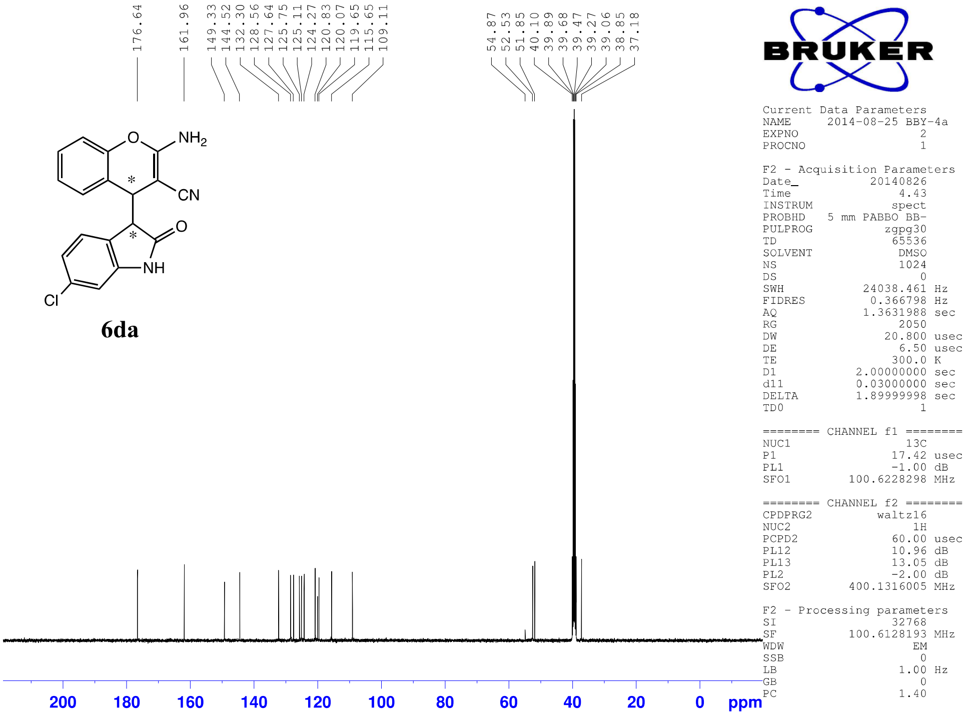

Figure S15. H-NMR of 6db.

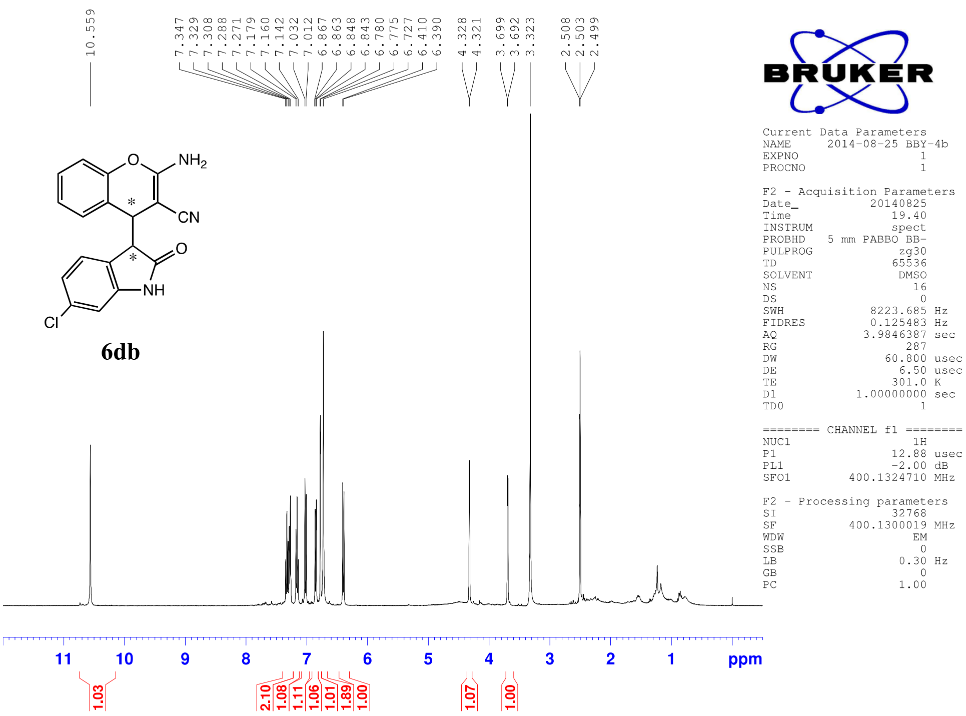

Figure S16. C-NMR of 6db

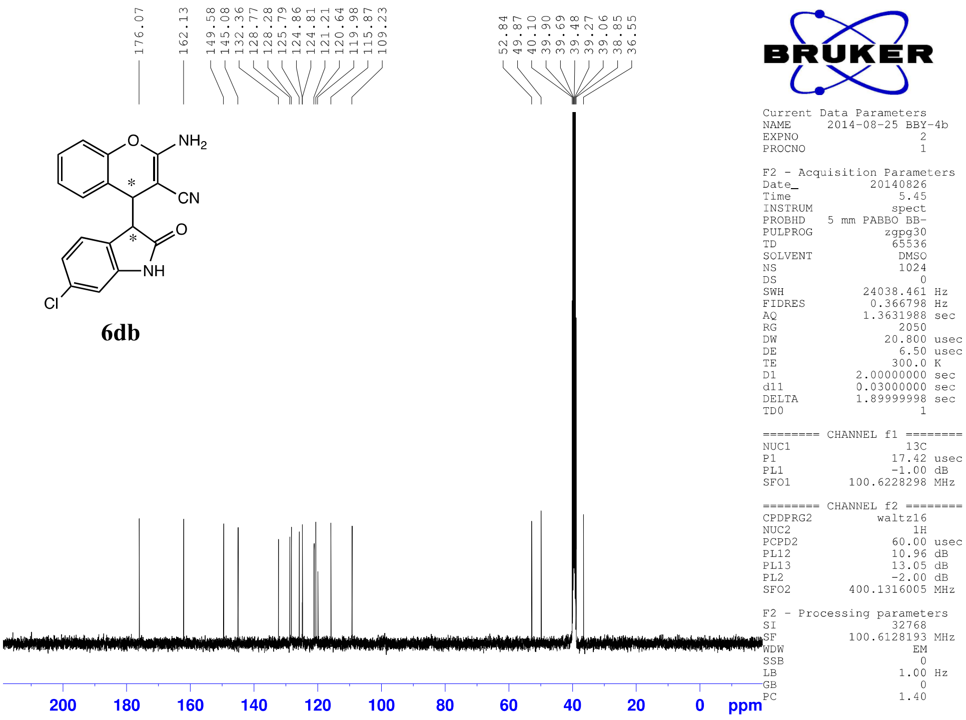

Figure S17. H-NMR of 6ea.

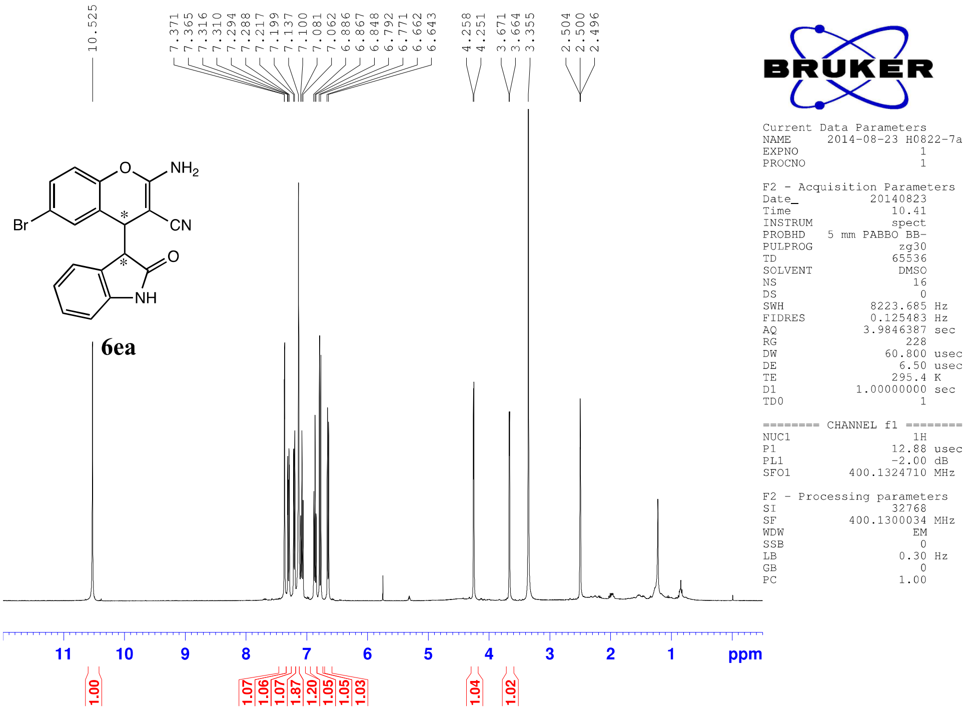

Figure S18. C-NMR of 6ea.

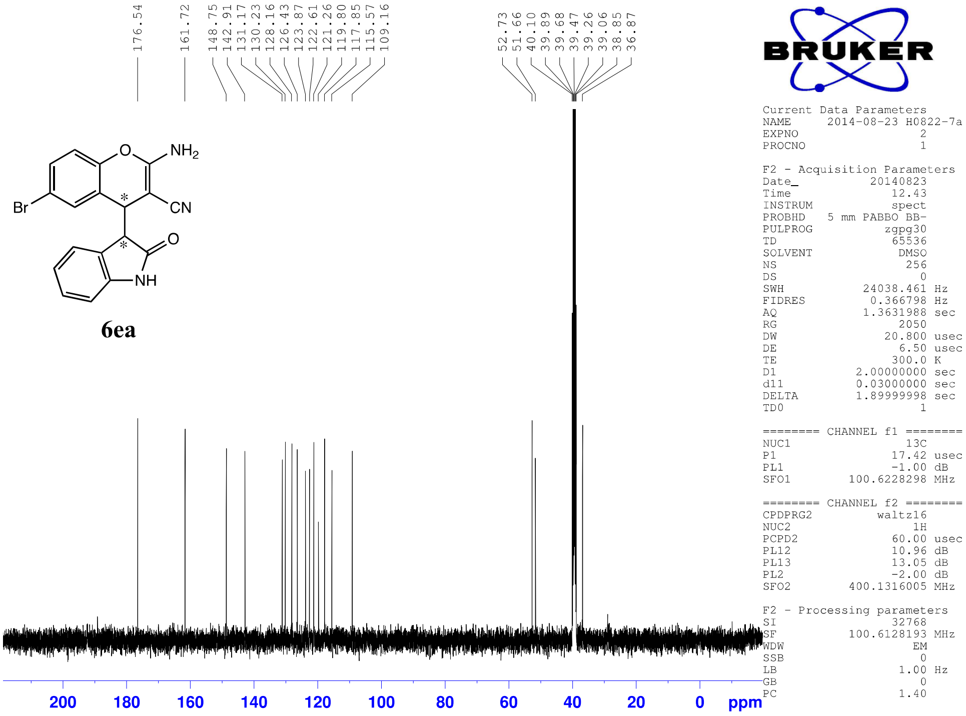

Figure S19. C-NMR of 6eb.

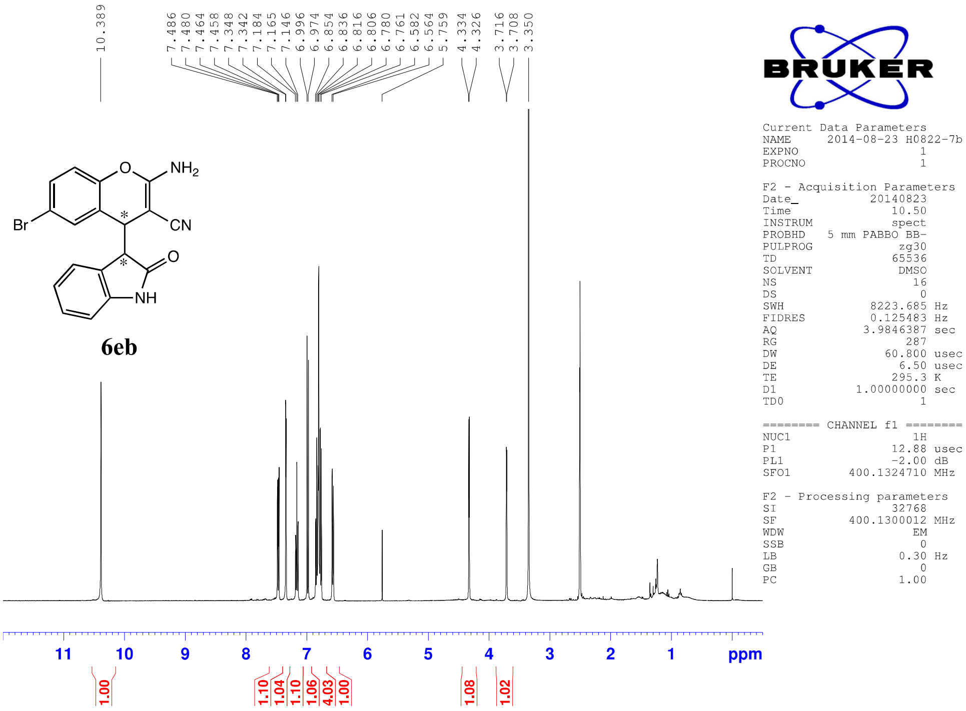

Figure S20. C-NMR of 6eb.

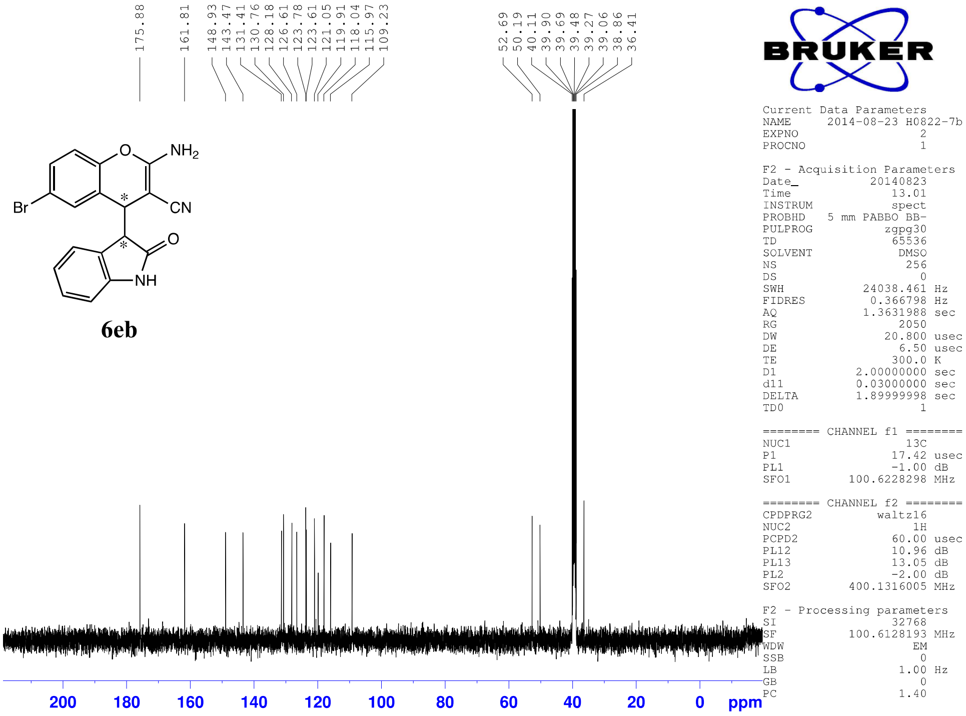

Figure S21. H-NMR of 6fa.

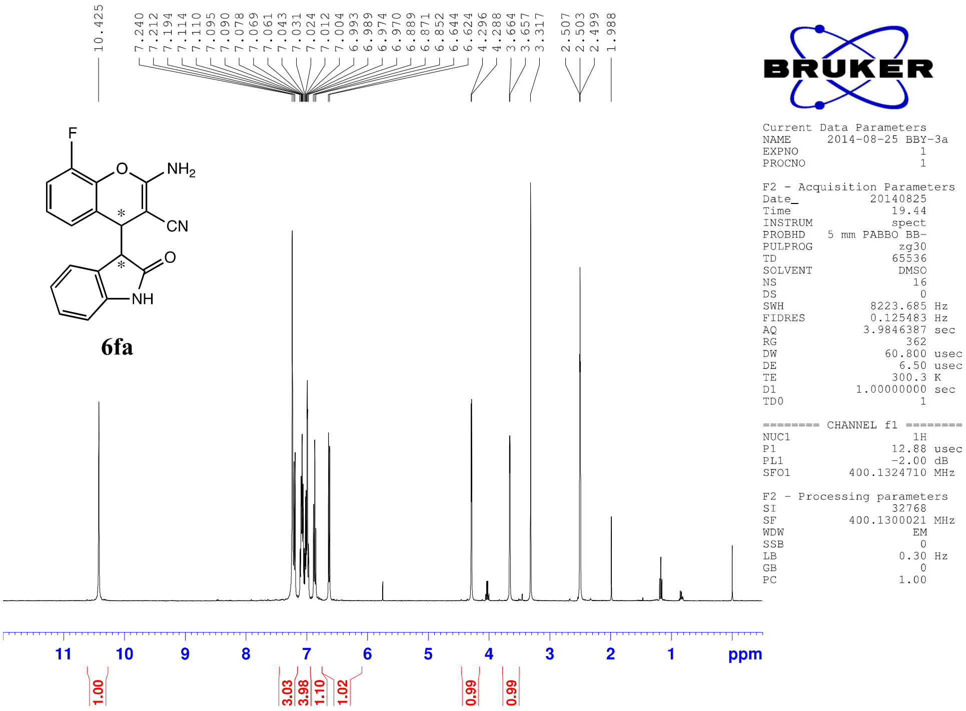

Figure S22. C-NMR of 6fa.

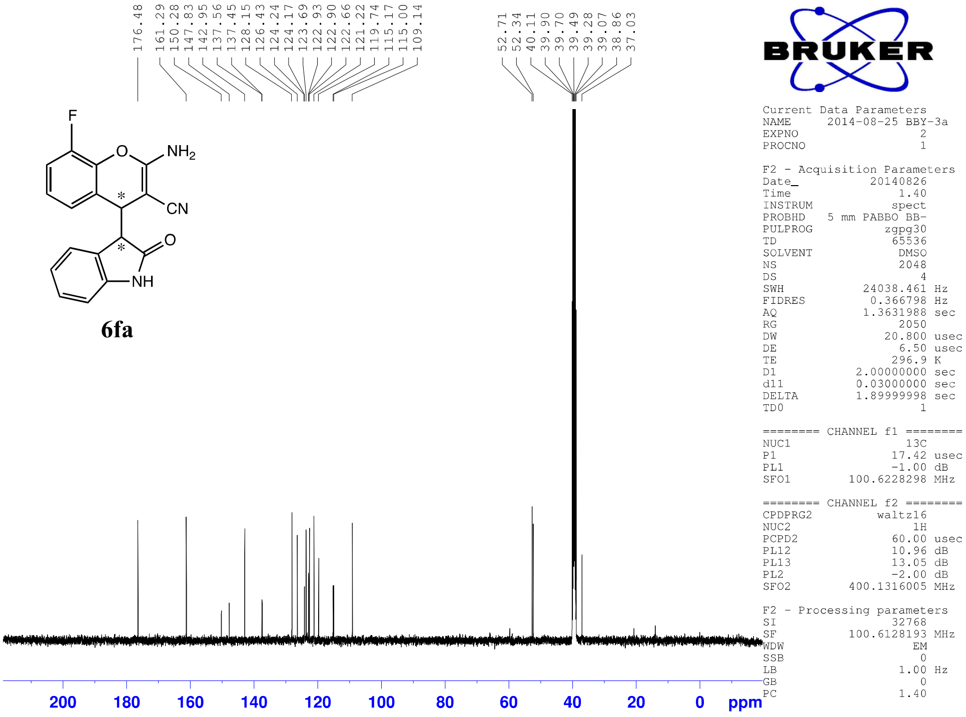

Figure S23. H-NMR of 6fb.

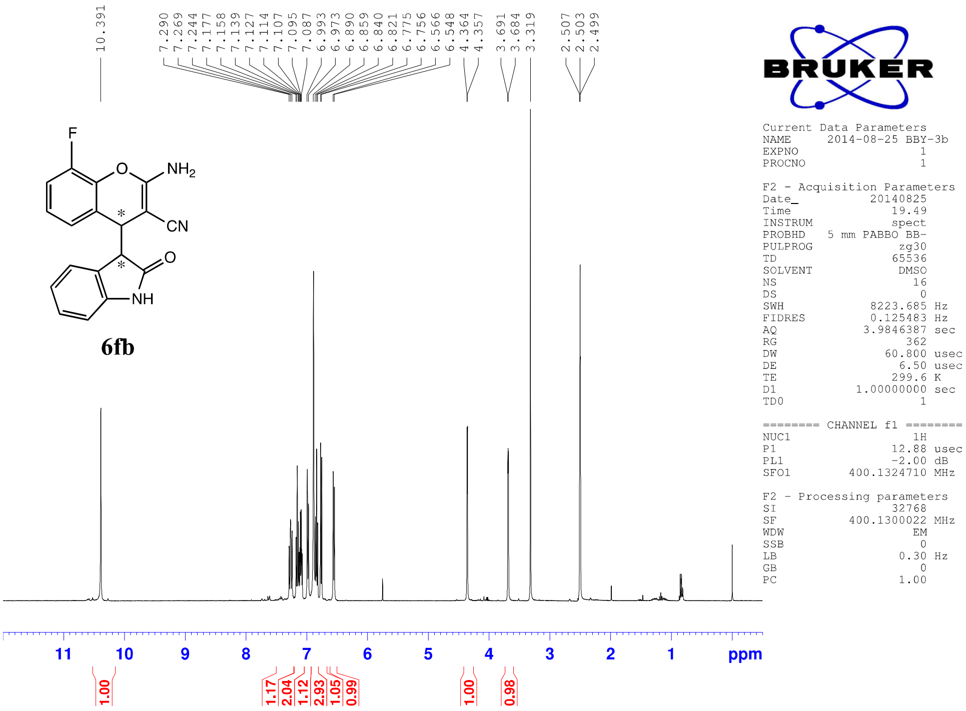

Figure S24. C-NMR of 6fb.

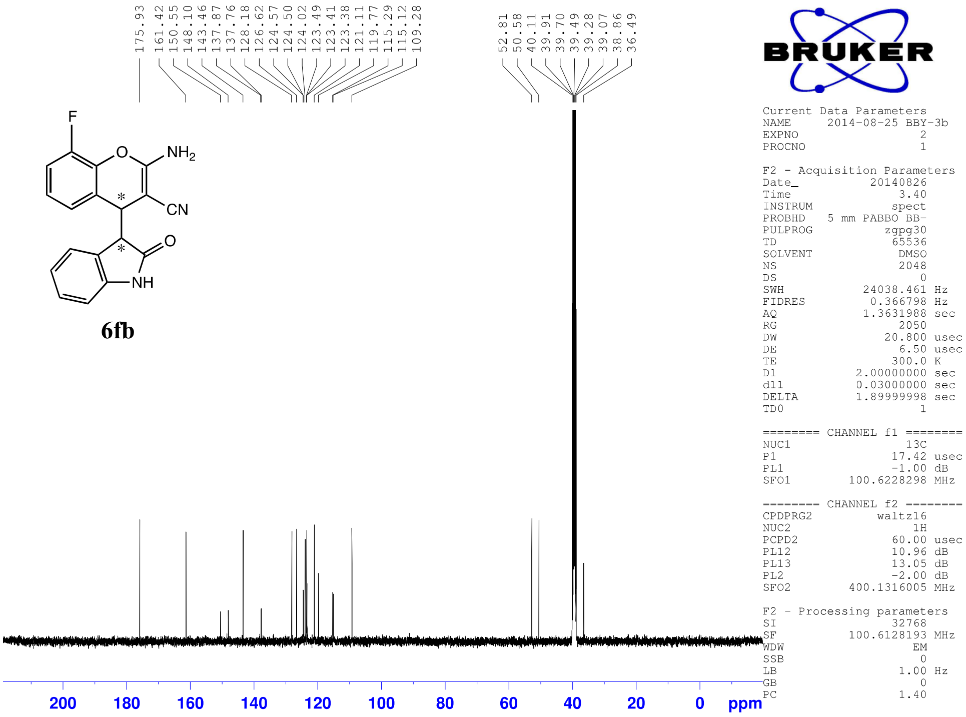

Figure S25. H-NMR of 6ga.

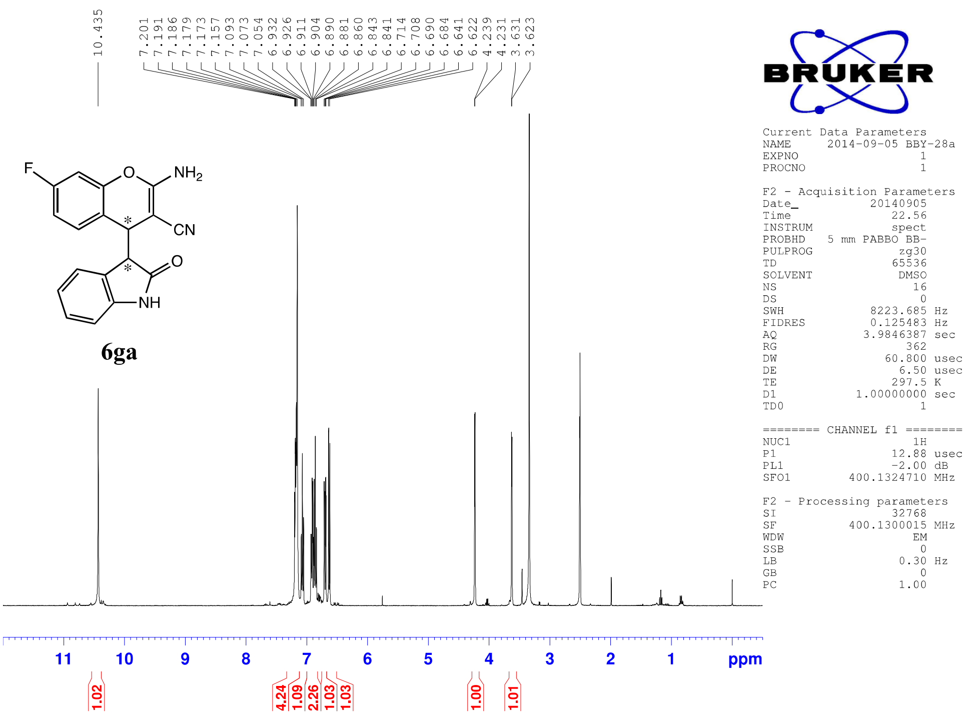

Figure S26. C-NMR of 6ga.

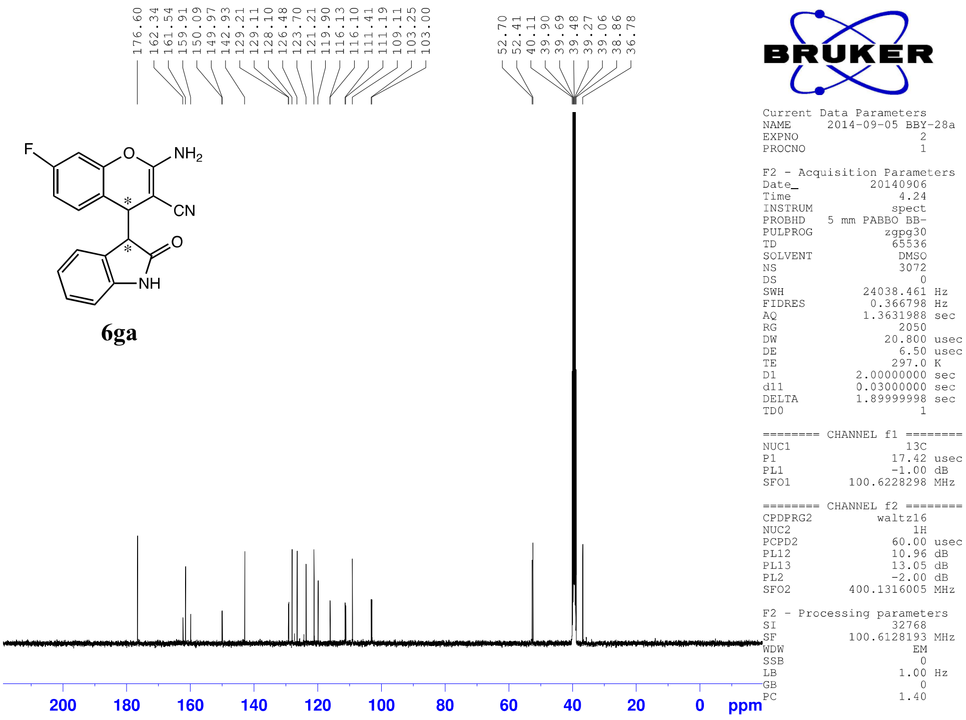

Figure S27. H-NMR of 6gb.

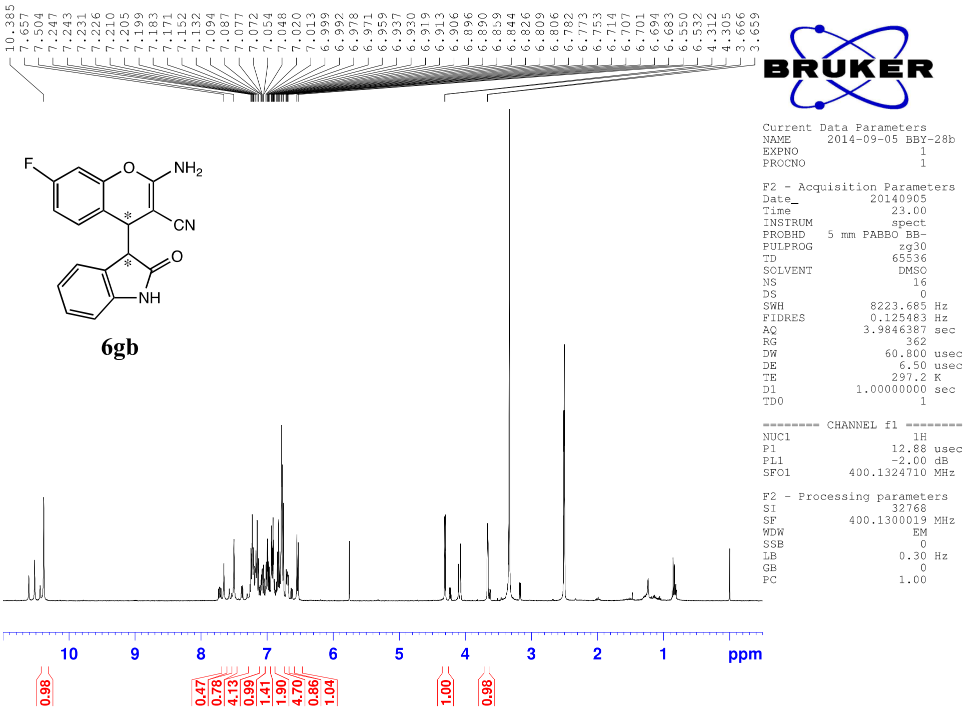

Figure S28. C-NMR of 6gb.

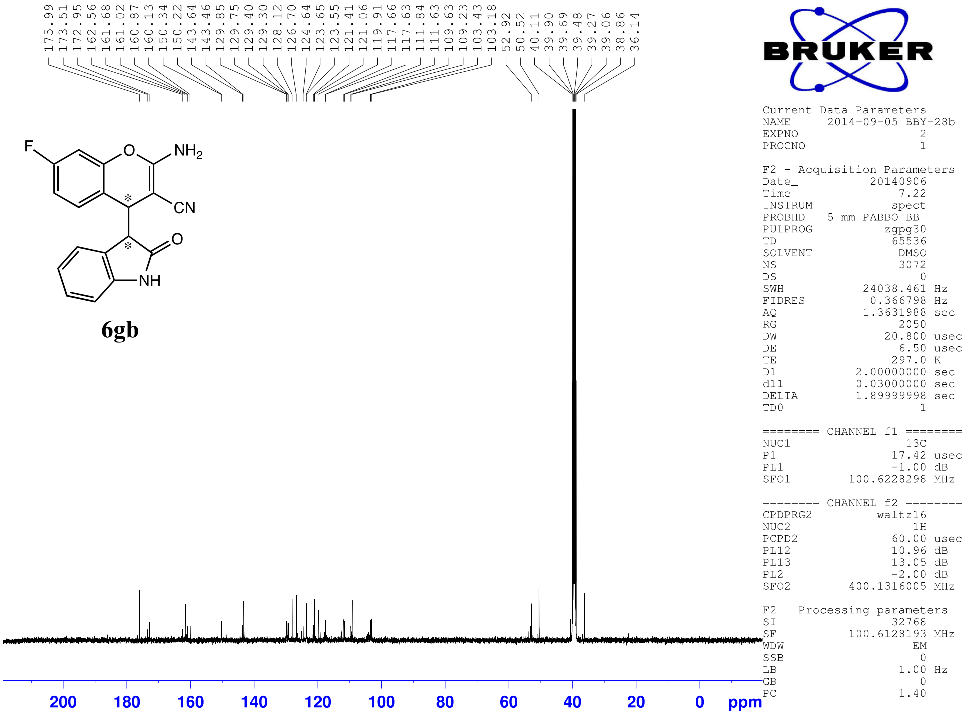

Figure S29. H-NMR of 6ha.

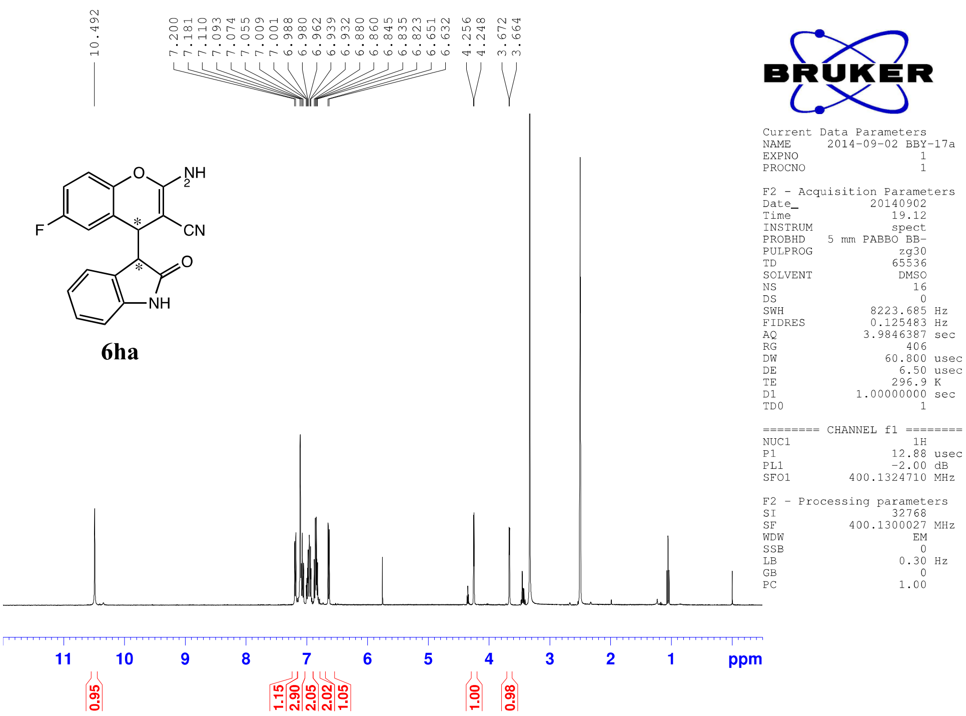

Figure S30. C-NMR of 6ha.

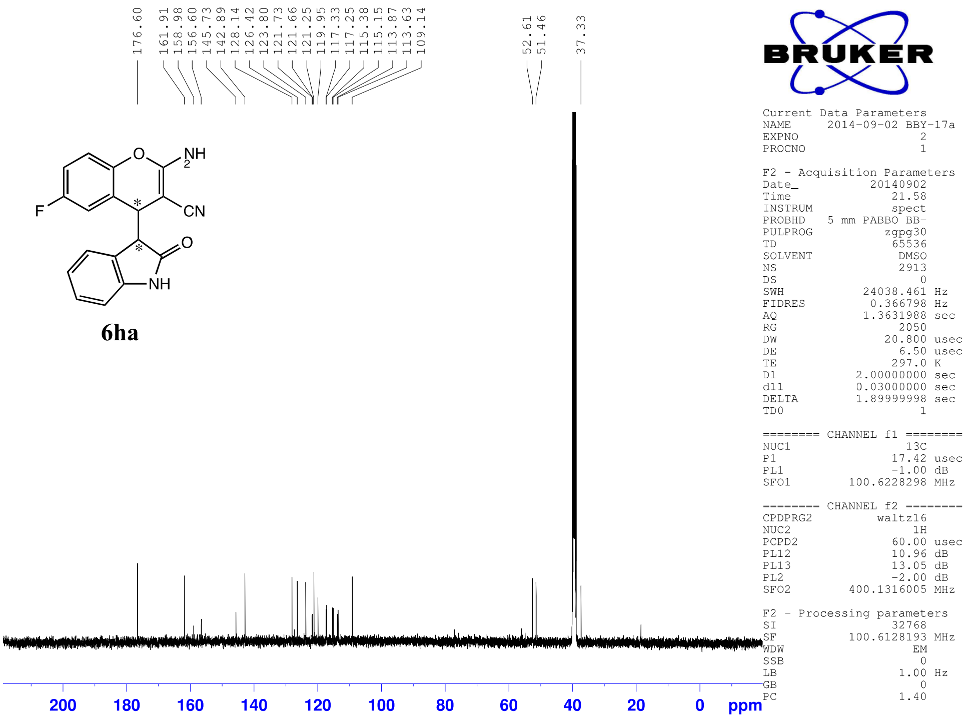

Figure S31. H-NMR of 6hb.

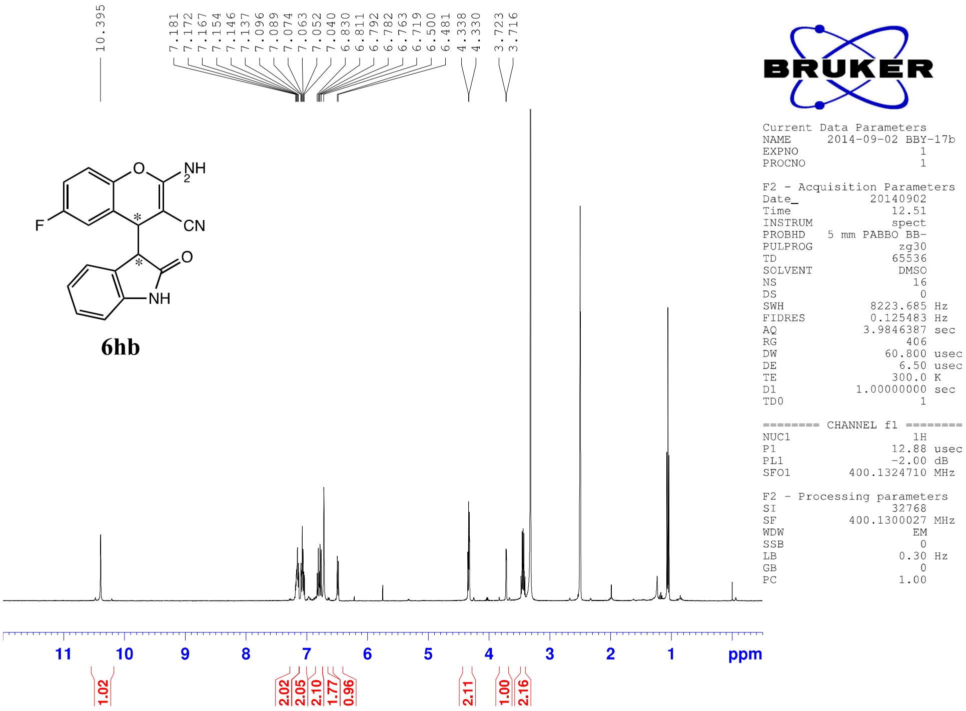

Figure S32. C-NMR of 6hb.

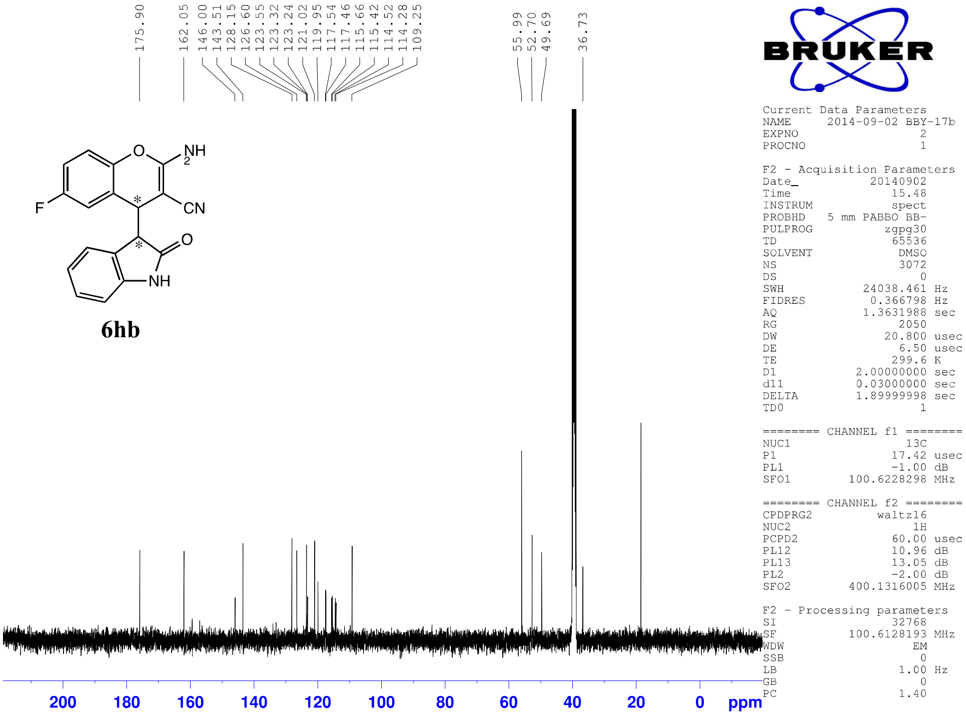

Figure S33. H-NMR of 6ia.

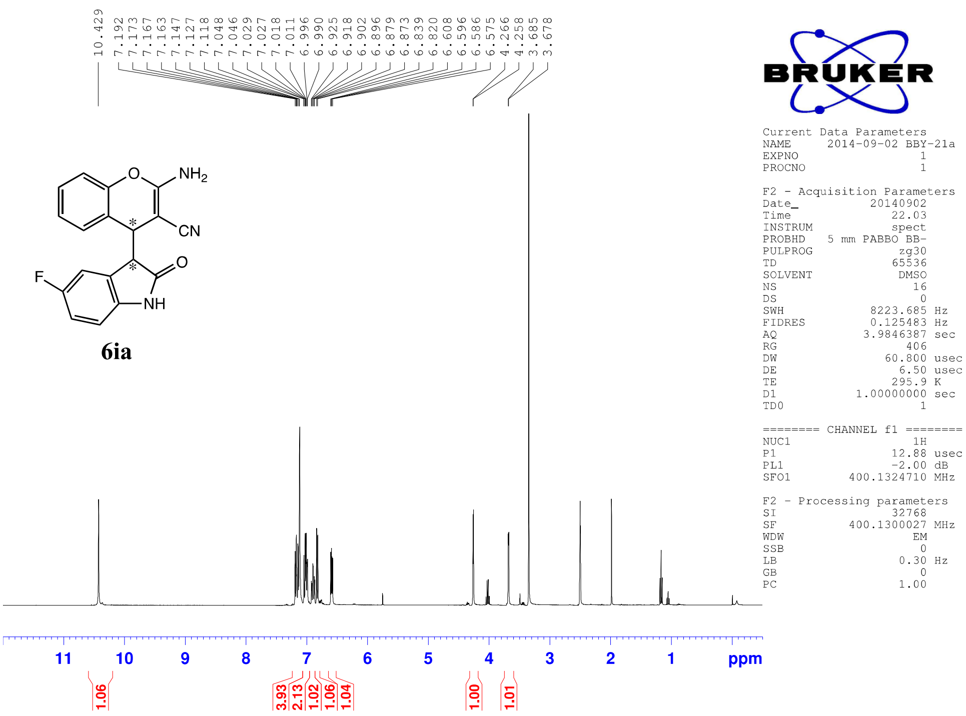

Figure S34. C-NMR of 6ia.

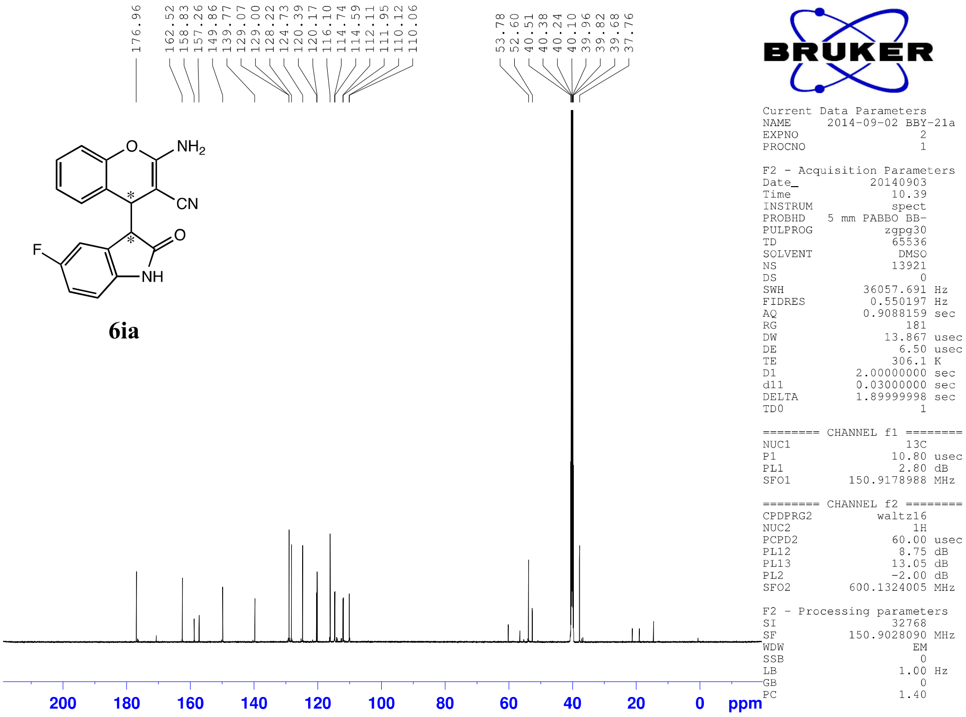

Figure S35. H-NMR of 6ib.

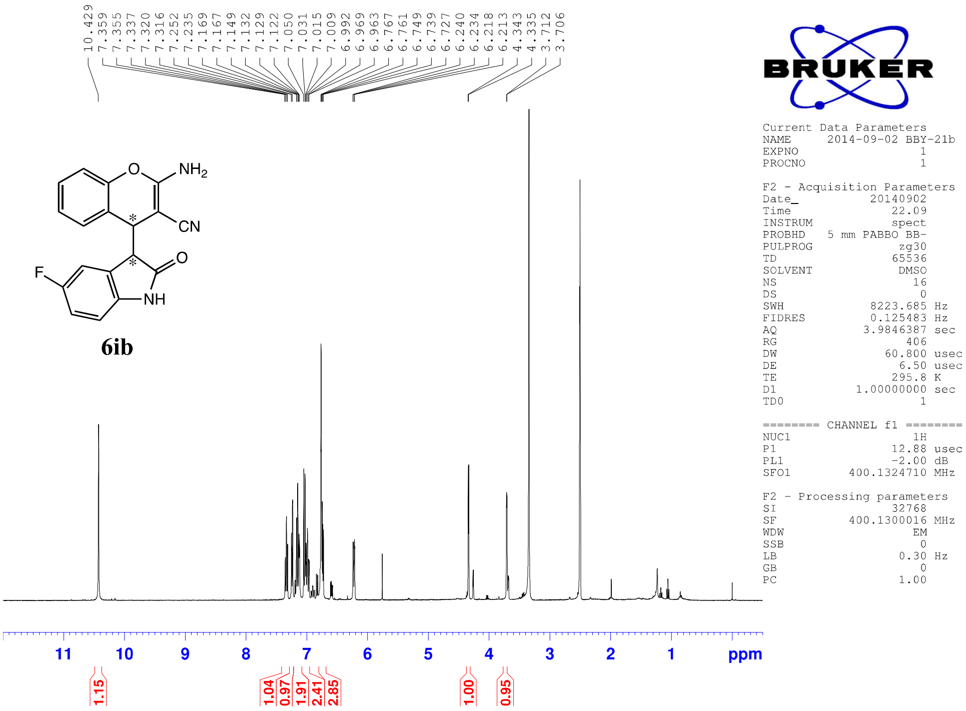

Figure S36. C-NMR of 6ib.

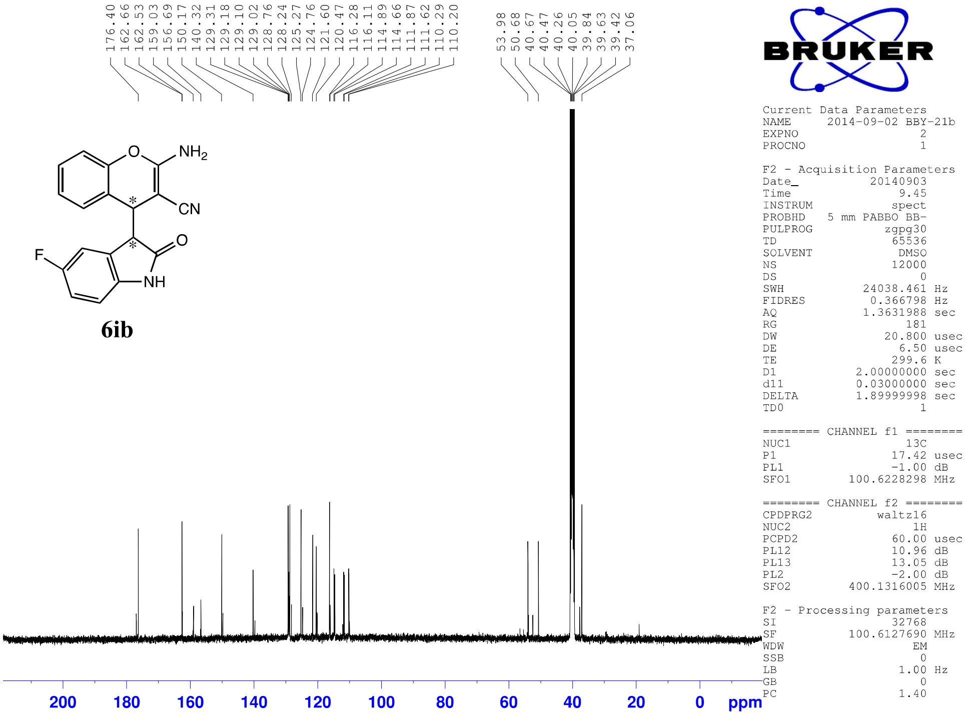

Figure S37. H-NMR of 6ja.

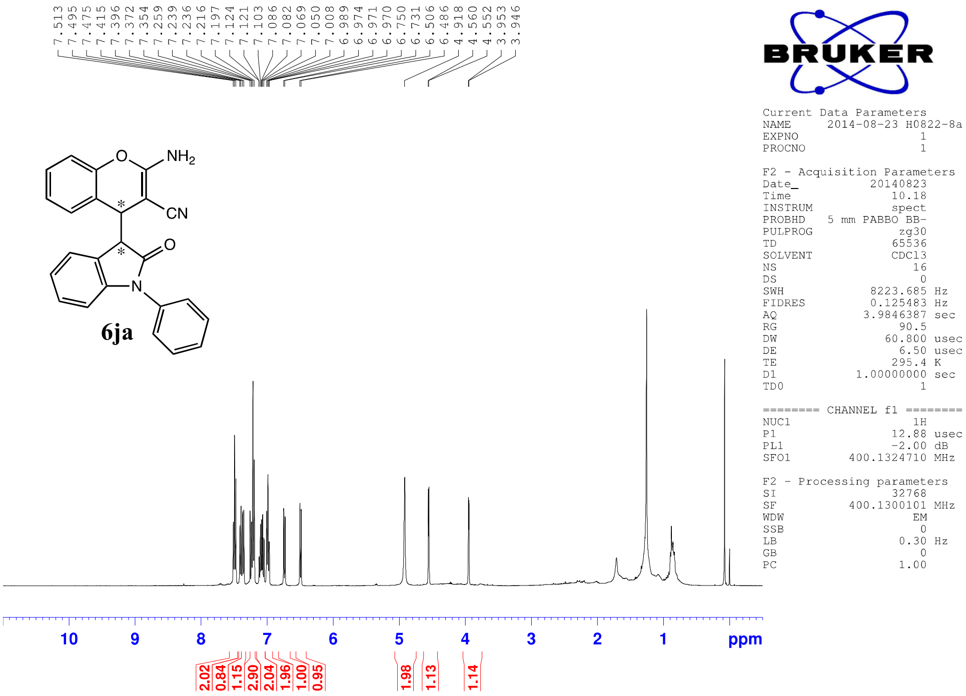

Figure S38. C-NMR of 6ja.

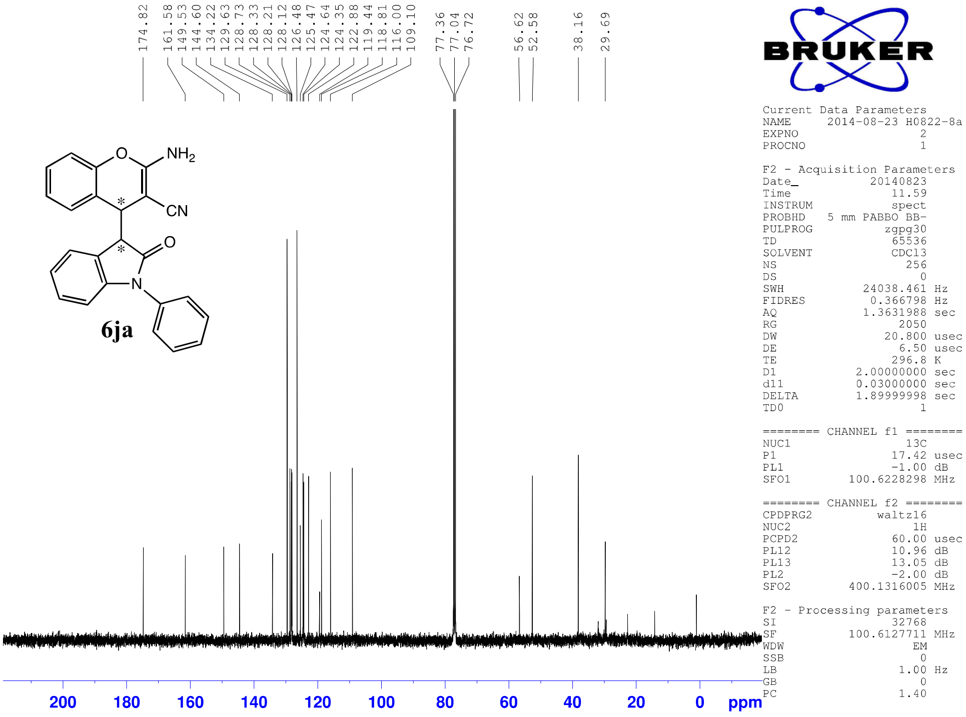

Figure S39. H-NMR of 6jb.

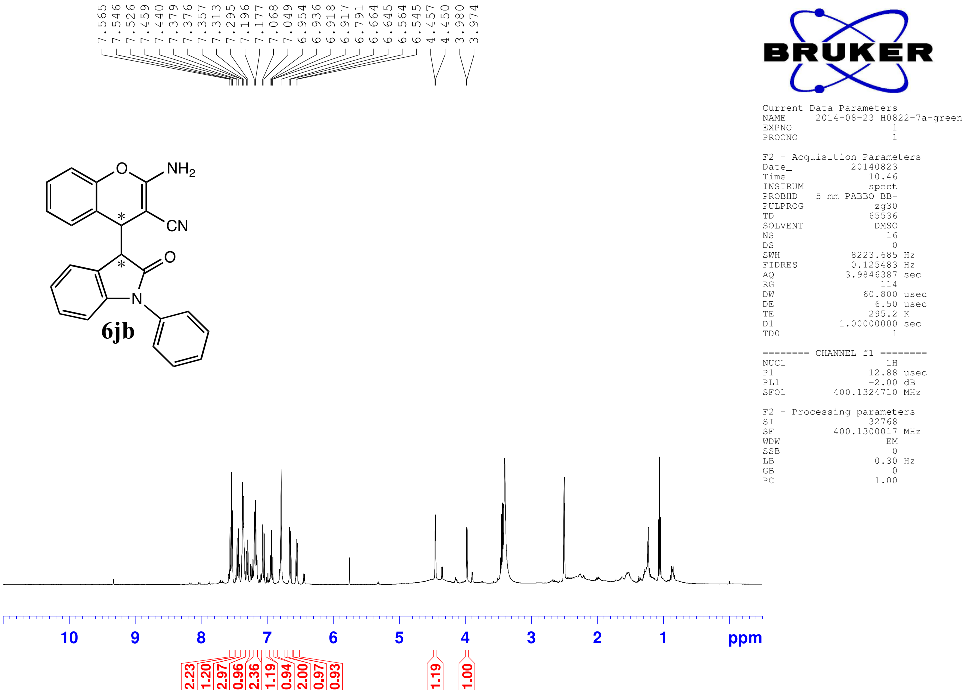

Figure S40. H-NMR of 6ka.

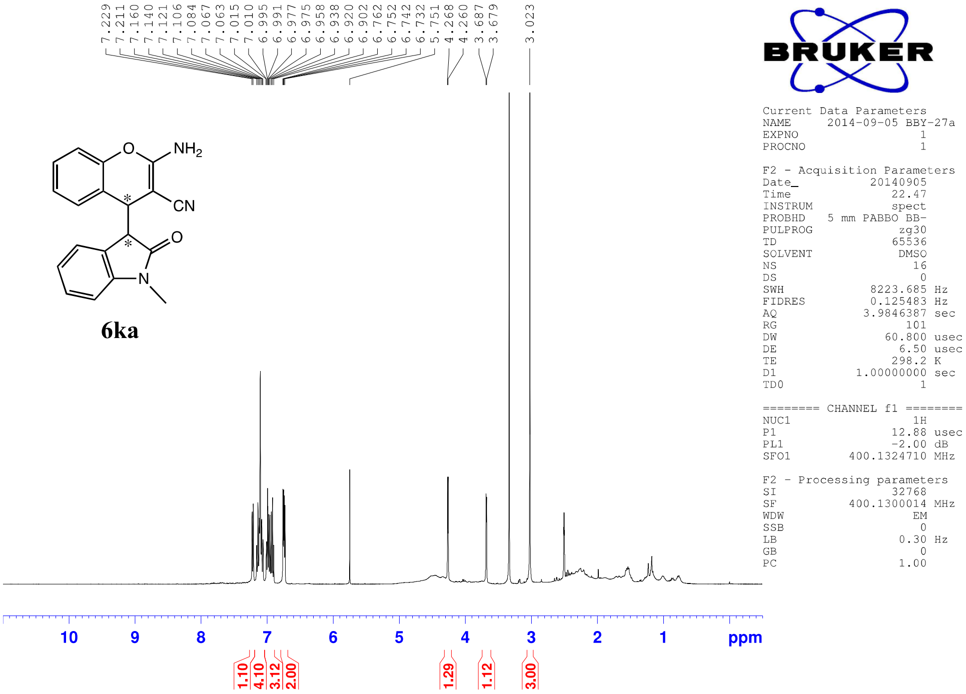

Figure S41. C-NMR of 6ka.

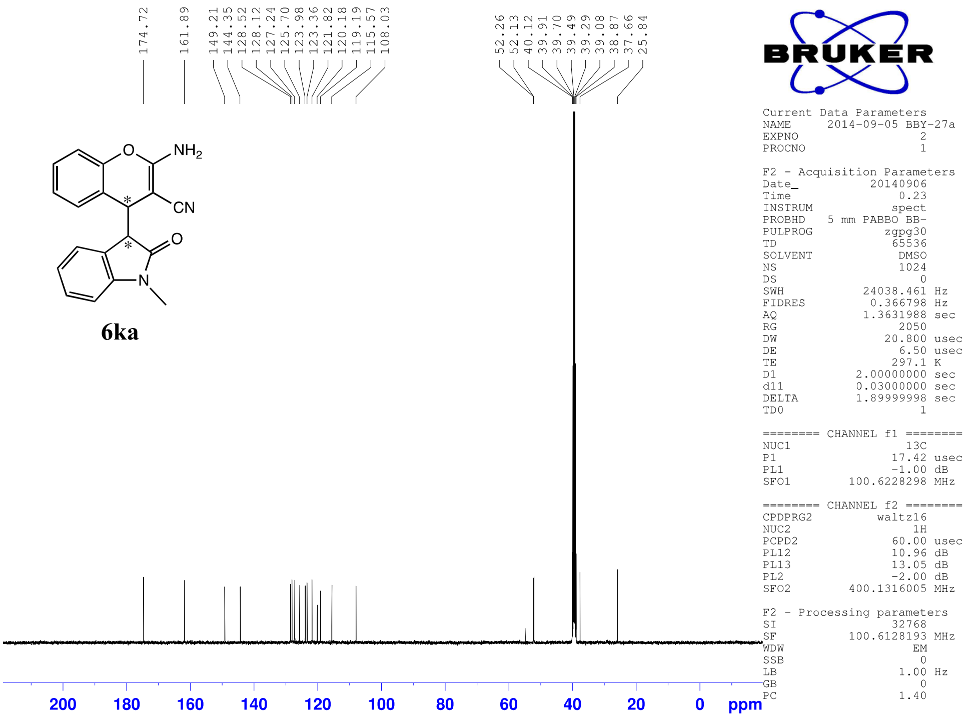

Figure S42. H-NMR of 6kb.

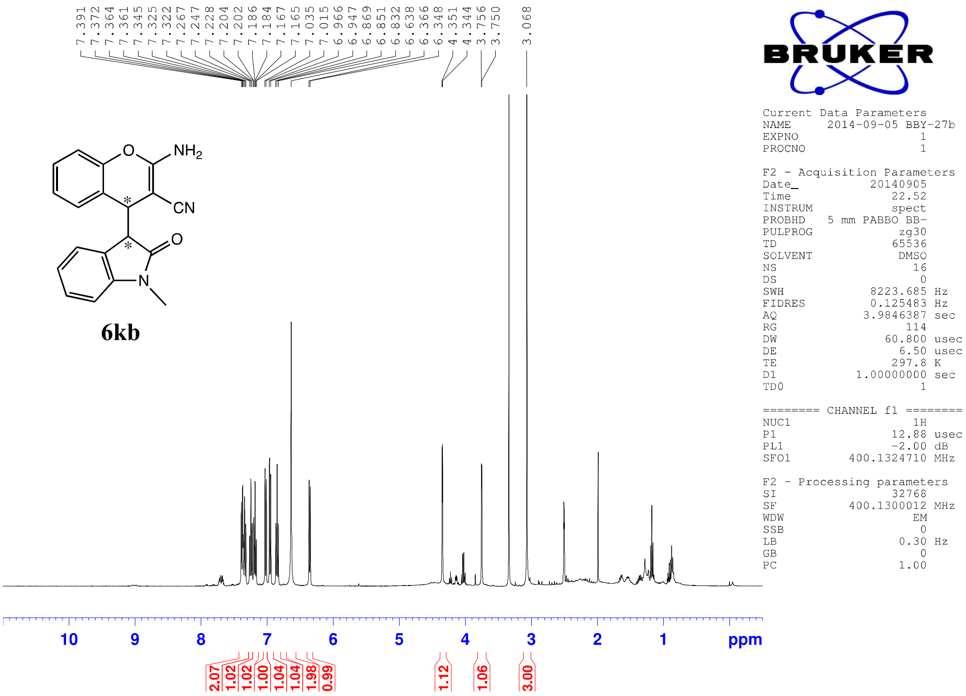

Figure S43. C-NMR of 6kb.

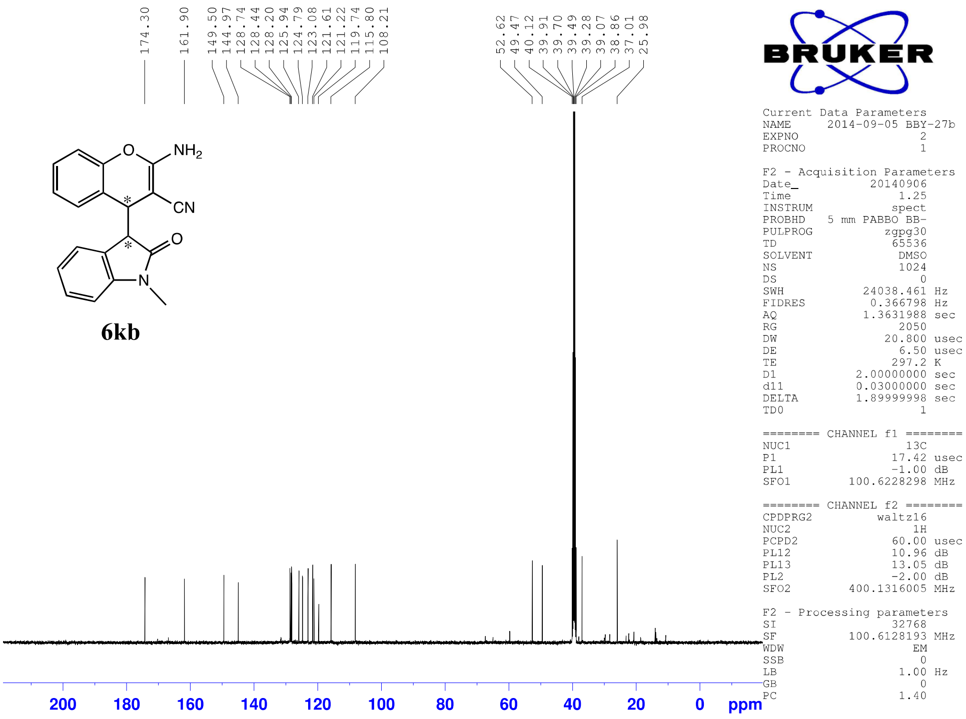

Figure S44. H-NMR of 6la.

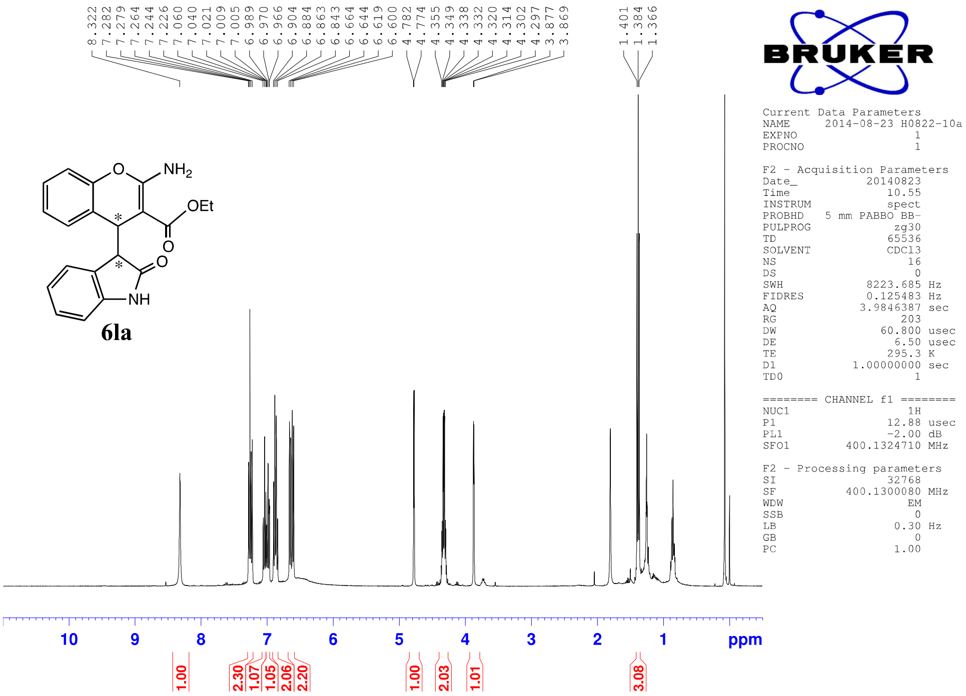

Figure S45. C-NMR of 6la.

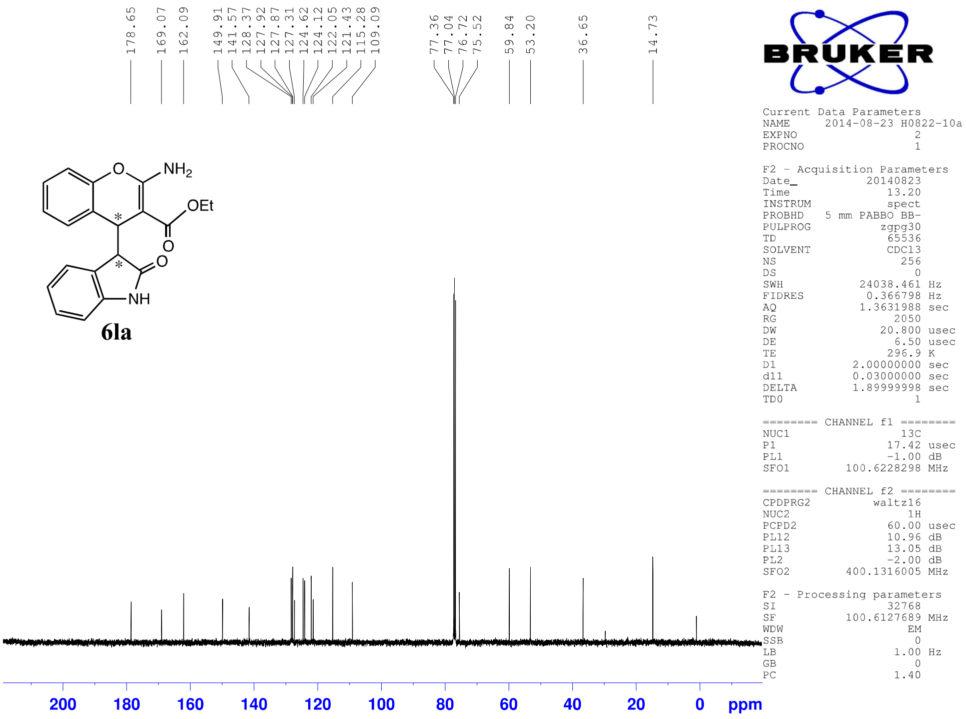

Figure S46. H-NMR of 6ma.

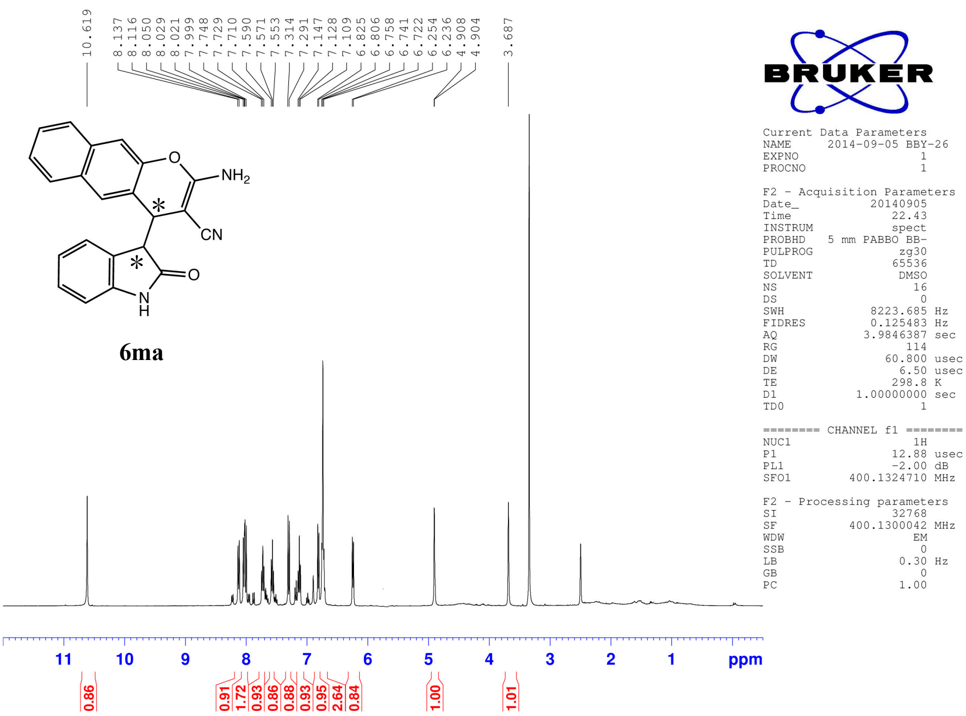

Figure S47. C-NMR of 6ma.

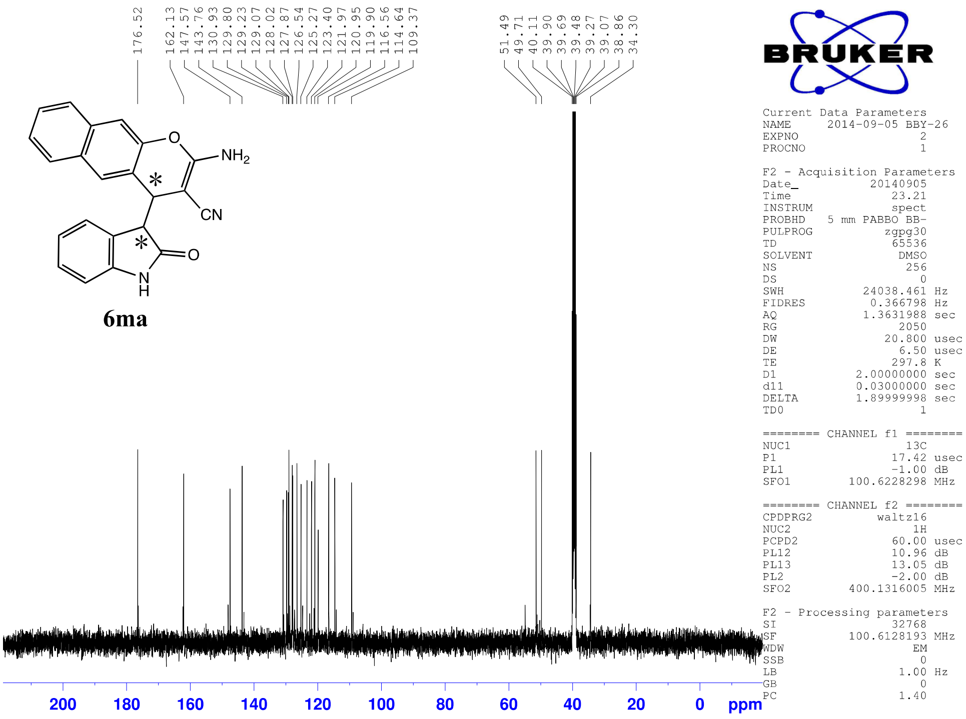

Figure S48. H-NMR of 6n.

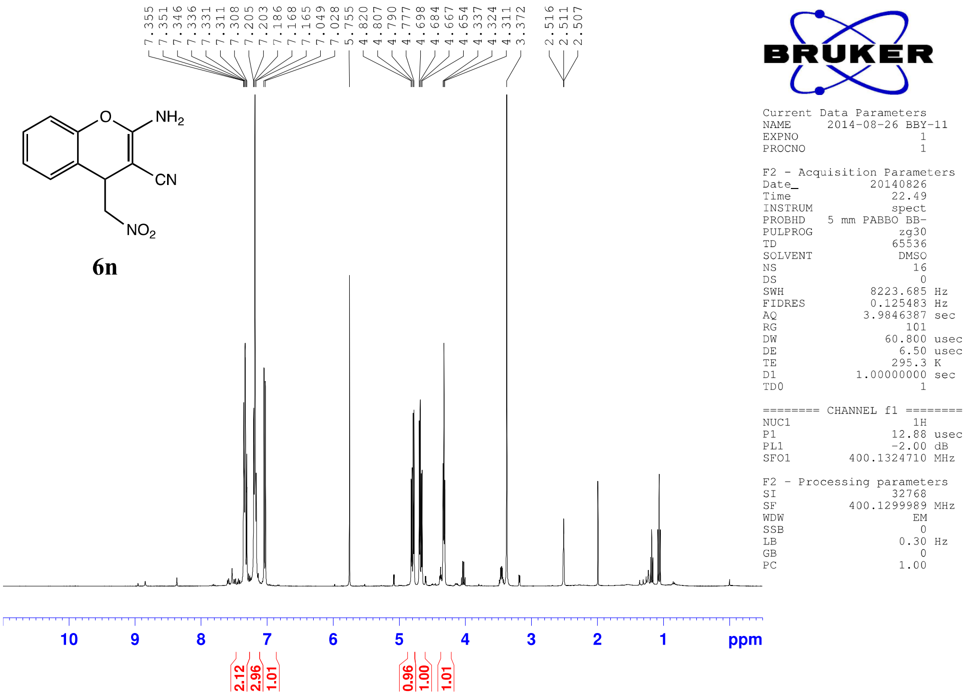

Figure S49. C-NMR of 6n.

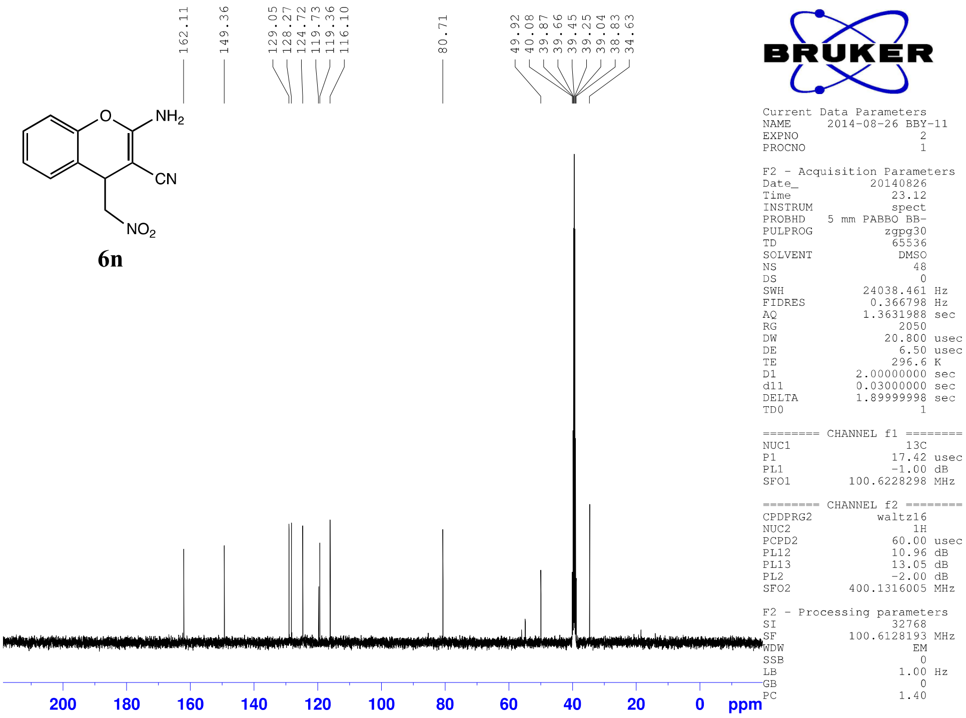

Figure S50. H-NMR of 6o.

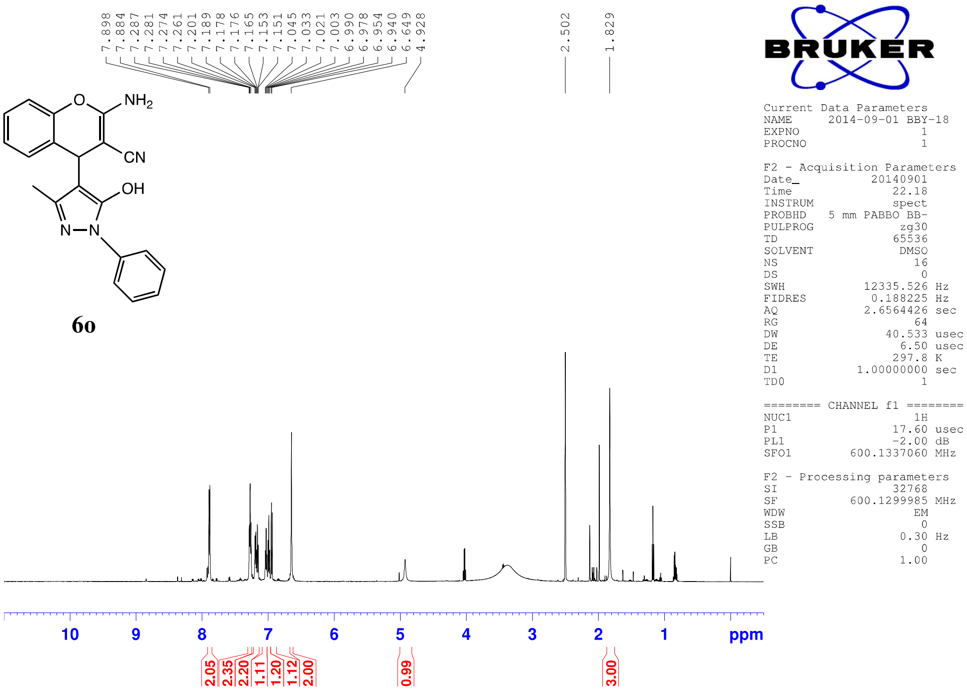

Figure S51. H-NMR of 6p.

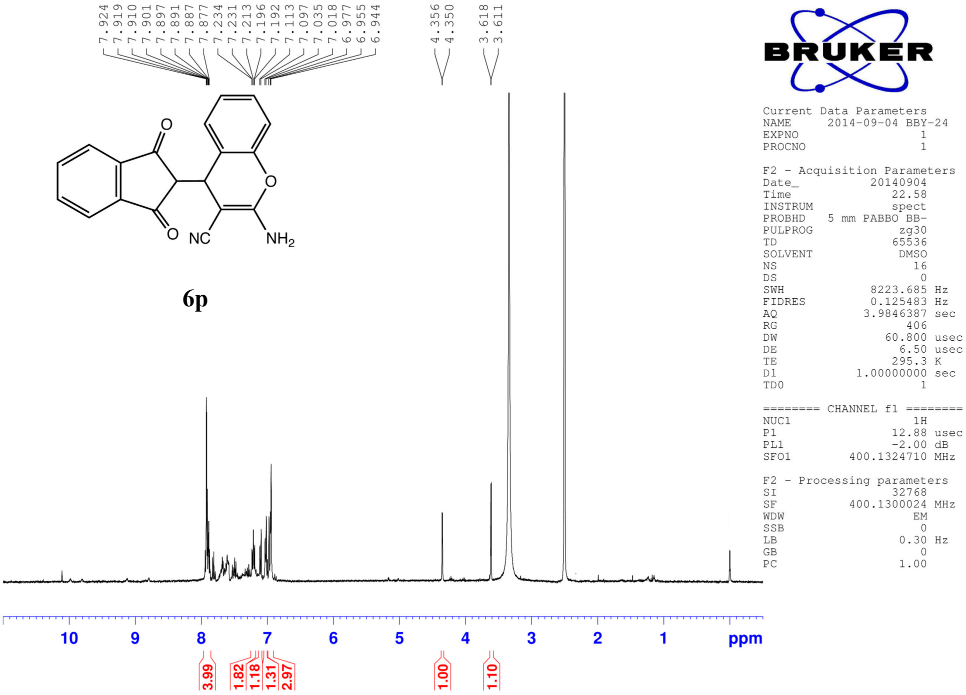

Figure S52. H-NMR of 6q.

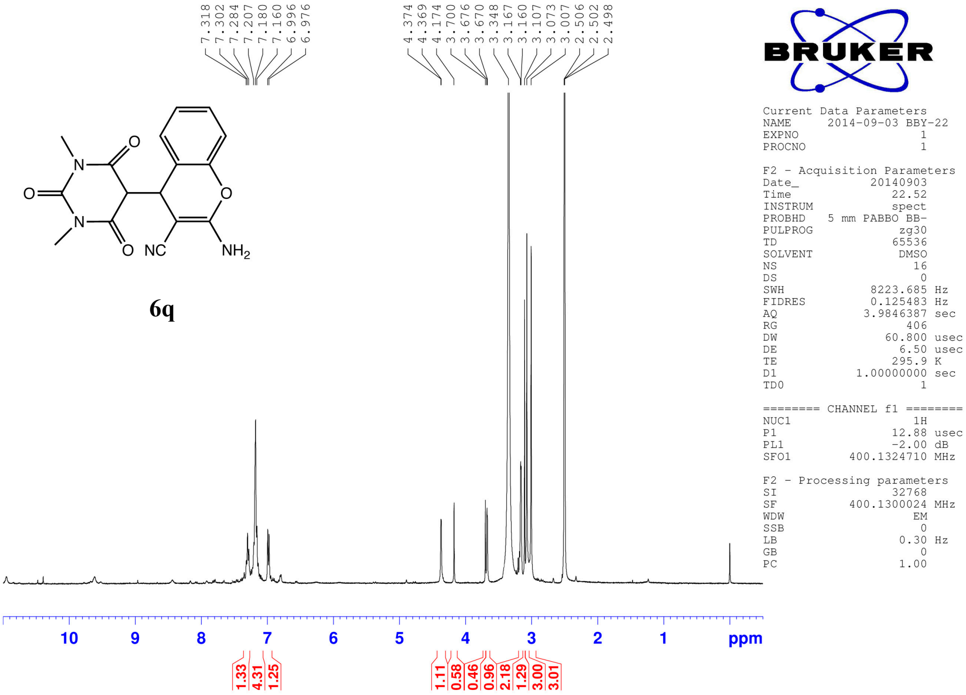

Supplement: Supplementary file 1 [file molecules-19-19253-s001.pdf]
